# Predictive model of surgical infection to enhance patient safety: A retrospective cohort study

**DOI:** 10.1590/1980-220X-REEUSP-2025-0207en

**Published:** 2025-12-01

**Authors:** Christiany Moçali Gonzalez, Joana de Oliveira Pantoja Freire, Camila Medeiros dos Santos de Cerqueira, Graciele Oroski Paes

**Affiliations:** 1 Universidade Federal do Rio de Janeiro, Escola de Enfermagem Anna Nery, Pós-graduação da Escola de Enfermagem Anna Nery, Rio de Janeiro, RJ, Brazil.

**Keywords:** Risk Assessment, Surgical Wound Infection, Patient Safety

## Abstract

**Objective:**

To identify risk factors for surgical site infection and to establish a prediction model.

**Method:**

Retrospective cohort study with 20,778 surgeries performed between 2009 and 2019 at a university hospital in Rio de Janeiro. Clinical and operative variables were analyzed using multivariate logistic regression (p≤0.05).

**Results:**

The overall surgical infection rate was 7.2%. Age ≥41 years presented an odds ratio between 1.52 and 3.77 (p < 0.0001). Contaminated and infected surgeries increased the risk threefold (95% CI: 2.48–3.63). Urgent procedures (OR = 2.04; 95% CI: 1.83–2.28) and ASA III (OR=3.77) were associated with a higher risk. Each additional hour of surgery increased the risk by 34% (OR = 1.34; 95% CI: 1.30–1.38). Conventional technique had a risk 2.7 times greater than videolaparoscopy (RC = 2.72; p < 0.0001).

**Conclusion:**

The developed model allows for precise stratification of surgical site infection risk and supports preventive strategies, improving the surveillance and management of surgical risk in highly complex hospitals.

## INTRODUCTION

Surgical site infections (SSIs) are complications related to surgical procedures, with or without implant placement, in inpatients or outpatients. They occur when there is at least one incision, performed in an operating room, made through the skin, mucous membrane, or an incision that was left open during a previous surgical procedure^(1)^.

These infections have a variable incidence and represent a major challenge in preventing healthcare-associated infections (HAIs), ranking third among all infections in healthcare services and comprising, in Brazil, 14% to 16% of HAIs found in hospitalized patients^(2)^.

In high-income countries like the United States of America, this issue also remains relevant. Recent data indicate that SSIs account for more than two million nosocomial infections in hospitalized patients, corresponding to 20% of major infections, highlighting the severity of the problem even in advanced health systems^(3–4)^. In Europe, the situation is no different, it is considered the second most common infection, accounting for around 500,000 cases per year^(5)^.

Increased length of hospital stay, up to three times^(5–6)^, increased hospital costs, and a 2 to 11 times greater risk of death compared to patients who do not develop SSI^(3,7–8)^ are significant impacts that not only overload hospital systems, but also increase operating costs, limit bed availability for other patients, and require priority attention in the healthcare context^(9–10)^.

These factors affect not only hospitals, but also patients themselves, who face greater suffering, postoperative complications and socioeconomic impacts, such as prolonged absence from work^(7)^. In light of these facts, the work of a multidisciplinary team is crucial for implementing rigorous surveillance strategies and infection control, and improving surgical practices, therefore being essential for mitigating these risks and ensuring better clinical outcomes^(11)^.

Given the impacts presented and considering their preventability, it is essential to implement preventive measures to mitigate the risks of this event occurring in health institutions, as directed by the international guidelines of the World Health Organization (WHO) and the national guidelines of the Brazilian Health Regulatory Agency (ANVISA).

The implementation of these strategies reflects a global commitment to patient safety and the continuous improvement of surgical care, as these measures aim not only to reduce SSI rates but also to minimize the risks of serious complications, ensuring the quality of care and promoting safer surgical procedures.

In this context, the nurse has a prominent role, since this professional is responsible for planning and implementing the necessary nursing care and health education. Furthermore, they must have knowledge about complications, aimed at their prevention and early detection, effectively assisting the patient’s well-being and new living conditions^(12)^.

Therefore, it is imperative that we have a solid understanding of the relationship between risk factors and SSI outcomes, as these factors are seen as surrogates for the underlying cause and are used to predict outcomes. When a factor is repeatedly shown to be associated with SSIs, it becomes a more reliable indicator for the development of prevention and surveillance strategies. Additionally, consistent risk factors can be used to standardize SSI rates across patients, allowing for more accurate comparisons and contributing to quality improvement initiatives^(7)^.

In view of the above, studies with comprehensive analysis of a large cohort of patients, together with the identification of risk and protective factors, increase our understanding of this complex surgical complication. Understanding and stratifying this risk is essential to improve outcomes, guide surveillance decisions, and allocate resources efficiently, as these rates themselves are difficult to interpret without risk stratification^(7,13)^. Therefore, a risk stratification tool is a key element in the diagnostic pathway and allows for the early identification of individuals with high risk factors, enabling the implementation of more effective monitoring and prevention strategies, aiming at continuous improvement in quality and patient safety.

In this circumstances, this study aims to identify the risk factors for surgical site infection in surgeries performed in a university hospital and to establish a prediction model.

## METHOD

### Design of Study

This is a quantitative, observational, retrospective cohort study.

### Local

The location chosen to develop the study was the Federal University Hospital, a reference in the treatment of various highly complex pathologies and an important center for assistance, teaching, research, and extension, located in Rio de Janeiro.

### Study Sample

The sample used in the research was extracted from the surgical surveillance database, belonging to the Hospital Infection Control Coordination (*CCIH*), linked to the hospital’s electronic medical record system, covering the period from 2009 to 2019. The time frame adopted in this research was defined to ensure data consistency and comparability. The years 2020 and 2021 were excluded due to the direct impact of the COVID-19 pandemic on surgical flows and hospital dynamics. During this period, there was a significant reduction in the performance of elective procedures, prioritization of urgent and emergency surgeries, and restructuring of health services aimed at tackling the health crisis.

These changes compromised the regularity of surgical practices and the reporting and surveillance processes for healthcare-associated infections, especially with regard to SSI. As a consequence, the data from these years present atypical characteristics, which could introduce biases into the statistical analysis and hinder the identification of epidemiological patterns and risk factors in stable care contexts. The research included 20,778 surgeries.

### Selection Sriteria

The study included data from surgeries that met the criteria established by ANVISA: those surgeries whose patient’s hospital discharge date differed from the date of the procedure and those in which the patients were 18 years of age or older. Reoperations performed less than 30 days after the first intervention, surgeries performed in hemodynamics, and surgical procedures indicated for the treatment of acute trauma were excluded.

### Data Collection

Data collection took place from October 2023 to April 2024.

### Data Analysis

Surgical site infection in surgical procedures and its possible risk factors were analyzed. The categorization of SSI was based on the absence and presence of infection, which could be incisional, superficial or deep, of organs and spaces.

The independent variables considered were the risk factors for SSI, those whose presence is associated with a greater probability of an infection development: sex; potential for contamination of the surgical wound (clean, potentially contaminated, contaminated, and infected); patient’s clinical conditions in the preoperative period, analyzed by ASA I, II, III, IV and V, criteria proposed by *American Society of Anesthesiologists*; emergency (no and yes); implant (no and yes); type of surgical procedure; and duration of surgery.

The analyses were performed using descriptive statistics, with calculation of mean, median and standard deviation (SD) for quantitative variables. Qualitative variables were described based on absolute (n) and relative (%) frequency. Bivariate analyses including qualitative or categorical variables comprised the chi-square test and odds ratio.

For bivariate analyses, the normality of quantitative or continuous variables was first assessed using the Shapiro-Wilk test. Since the data did not present a normal distribution, the nonparametric Mann-Whitney U test was used to verify the association between demographic and clinical variables and the outcome.

In the multivariate logistic regression analysis, the backward method was used, that is, at each step, variables that were not significantly associated with the outcome were removed from the model. Thus, at the end of the multivariate model, those variables significantly associated with the presence of postsurgical infection remained.

Similarly, the association between demographic and clinical variables and hospital infection was verified through bivariate analyses. A significance level of 5% (p ≤ 0.05) and a 95% confidence interval were assumed for the analyses. All analyses were performed using IBM SPSS (v.16.0) and Microsoft Excel was used to prepare the figures.

### Ethical Aspects

The study was approved by the Research Ethics Committee (*CEP*), with opinion no. 6,231,690, in compliance with Resolution no. 466/2012 of the National Health Council (*CNS*). As this is a retrospective study carried out using secondary sources inherent to the HAI surveillance and control process already carried out by the *CCIH* at the study site, the signing of the Informed Consent Form was waived to carry out the study.

## RESULTS

The study included the analysis of 20,778 elective and emergency surgeries, distributed over 10 years of accounting. It was observed that the years 2013 (n = 2119; 10.2%) and 2016 (n = 2106; 10.1%) were those with the highest prevalence of surgeries performed.

Most procedures were performed on female patients (58.8%). Patients’ age ranged from 18 to 97 years, with a mean of 52.0 years (SD = 16.3 years) and a higher prevalence of surgical events (23.0%) for the age group of 51 to 60 years (Table 1).

**Table 1:** Descriptive analyses of demographic and clinical variables of patients undergoing surgical procedures between 2009 and 2019 (N = 20778) – Rio de Janeiro, RJ, Brazil, 2023–2024.

Variables	N	%	Min-Max	Mean (±SD)
Sex				
Male	8622	41.5		
Female	12156	58.5		
Age			18–97	52.0 (±16.3)
Age range				
From 18 to 30 years old	2551	12.3		
31 to 40 years	2746	13.2		
41 to 50 years	3819	18.4		
51 to 60 years	4875	23.5		
61 to 70 years	4017	19.3		
71 or older	2770	13.3		
Duration in hours			0.083–23.3	2.5 (±1.6)
ASA				
1	6273	30.2		
2	10375	49.9		
3	3818	18.4		
4	305	1.5		
5	6	0.0		
Type of surgery				
Elective	19390	93.3		
Urgent	1388	6.7		
Outcome				
Death	634	3.1		
Discharge	20144	96.9		
Use of Video				
No	17607	84.7		
Yes	3171	15.3		
Used implants				
No	15714	75.6		
Yes	5064	24.4		

Notes: SD = standard deviation; ASA = *American Society of Anesthesiologists*.

In men, the mean age was 53.03 (SD = 16.90) and in women it was 51.29 (SD = 15.78). The analysis of variance for sex in relation to age, using the Mann-Whitney technique, showed a violation of the null hypothesis (U = 5.62 × 10^7^; p < 0.001; VS-MPR = 8.51 × 10^15^; rbs = 0.07), indicating distinct patterns of age variation between the sexes.

The calculation of the duration of the surgeries performed showed that, on average, 2.46 hours (±1.60) were dedicated. Most procedures were elective (93.3%) and did not require reoperation (97.6%), while 6.7% were emergency surgeries. Another characteristic observed was ASA, whose most frequently observed classification was 2 (49.9%). Analysis of clinical outcomes revealed that 96.9% (n = 20,144) of patients were discharged from hospital, while 3.1% (n = 634) died. (Table 1)

The use of implants was recorded in 24.4% (n = 5,064) of the procedures, reinforcing the importance of these devices in surgical management, while 15.3% (n = 3,171) of the surgeries were video-assisted.

In the study sample, 1,491 occurrences of surgical site infection were recorded, resulting in an SSI incidence rate of approximately 7.2%. Of these, the majority (54.59%) were superficial and 31.52% were intracavitary. The place of infection was, for the most part, in the outpatient clinics for discharged patients (54.19%), as shown in Table 2.

**Table 2:** Characterization of the occurrence of Surgical Site Infection in participants over 10 years of observation (N = 20,778) – Rio de Janeiro, RJ, Brazil, 2023–2024.

Variables	N	%
Occurrence of infection		
Yes	1,491	7.18
No	19,287	92.82
Type of infection		
Superficial	814	54.59
Intracavitary	470	31.52
Deep	207	13.88
Locations of infection diagnosis		
Outpatient’s	808	54.19
Ward	683	45.81

Regarding sex, there was no statistically significant difference in the incidence of infection between men (7.0%) and women (7.4%) (OR = 1.05; 95% CI: 0.94–1.17; p = 0.374). The assessment of age group, in turn, revealed a significant association with the presence of infection (p < 0.0001). It was observed that, with advanced age, the chance of the outcome becomes greater in older groups (from 41–50 years old) when compared to younger groups (18–30 years old), as shown in Table 3.

**Table 3:** Types of surgical services and factors associated with Surgical Site Infection over 10 years of observation (N = 20778) – Rio de Janeiro, RJ, Brazil, 2023–2024.

Characteristics studied	Presence of infection				OR (95%CI)	p
	No		Yes			
	n	%	n	%		
Sex						
Male	11300	93.0	856	7.0	1.05 (0.94–1.17)	0.374
Female	7987	92.6	635	7.4		
Age range						
From 18 to 30 years old	2442	95.7	109	4.3	1.0	
31 to 40 years	2616	95.3	130	4.7	1.11 (0.86–1.45)	0.419
41 to 50 years	3576	93.6	243	6.4	1.52 (1.21–1.92)	<0.0001
51 to 60 years	4518	92.7	357	7.3	1.77 (1.42–2.21)	<0.0001
61 to 70 years	3655	91.0	362	9.0	2.22 (1.78–2.77)	<0.0001
71 or older	2480	89.5	290	10.5	2.62 (2.09–3.29)	<0.0001
Type of surgery						
Urgent	1209	87.1	179	12.9	2.04 (1.73–2.41)	<0.0001
Elective	18078	93.2	1312	6.8	1.0	
ASA						
Classification 1	6045	96.4	2299	3.6	1.0	
Classification 2	9629	92.8	746	7.2	2.04 (1.76–2.38)	<0.0001
Classification 3	3340	87.5	478	12.5	3.77 (3.20–4.44)	<0.0001
Contamination potential						
Clean	11130	94.7	626	5.3	1.0	
Potentially contaminated	5955	92.9	457	7.1	1.37 (1.21–1.55)	<0.0001
Contaminated	1726	84.2	323	15.8	3.33 (2.88–3.84)	<0.0001
Infected	476	84.8	85	15.2	3.18 (2.49–4.01)	<0.0001
Use of Video						
No	16245	92.3	1362	7.7	1.98 (1.64–2.38)	<0.0001
Yes	3042	95.9	129	4.1	1.0	
Use of implants						
No	13379	92.1	1146	7.9	1.47 (1.30–1.66)	<0.0001
Yes	5908	94.5	345	5.5	1.0	
Otorhinolaryngology						
Yes	1017	95.9	43	4.1	0.53 (0.38–0.72)	<0.0001
No	18270	92.7	1448	7.3	1.0	
Neurosurgery						
Yes	1215	92.5	99	7.5	1.06 (0.86–1.31)	0.603
No	18072	92.8	1392	7.2	1.0	
Urology						
Yes	1255	93.7	85	6.3	0.87 (0.69–1.09)	0.122
No	18032	92.8	1406	7.2	1.0	
Gynecology						
Yes	1507	92.1	129	7.9	1.12 (0.93–1.35)	0.247
No	17780	92.9	1362	7.1	1.0	
Plastic surgery						
Yes	1684	96.6	60	3.4	0.44 (0.34–0.57)	<0.0001
No	17603	92.5	1431	7.5	1.0	
Orthopedics and trauma						
Yes	3329	95.8	147	4.2	0.52 (0.44–0.62)	<0.0001
No	15958	92.2	1344	7.8	1.0	
General Surgery						
Yes	7246	91.9	638	8.1	1.25 (1.12–1.38)	<0.0001
No	12041	93.4	853	6.6	1.0	

Notes: OR = Odds Ratio; CI = Confidence Interval; ASA = American Society of Anesthesiologists.

Comparison of patients aged 31 to 40 years with the younger group did not reveal a significant association with the outcome (OR = 1.11; 95% CI: 0.86–1.45). However, for the following age group, the risk of SSI was 1.52 times higher compared to the same reference group (95% CI: 1.21–1.92). In summary, an increase in the chance of infection was observed with increasing age.

The potential for contamination during surgery was assessed in four categories, using the clean surgery classification as a reference. In comparison, it was observed that patients undergoing potentially contaminated procedures had a 37% greater chance of developing an infection. For contaminated procedures, the risk was 3.33 times higher (95% CI: 2.88–3.84). In infected procedures, the chance of developing the complication was 3.18 times higher (95% CI: 2.49–4.01).

People exposed to emergency surgeries had 2.04 times the chance (95% CI: 1.73–2.41) of presenting infection when compared to those undergoing elective surgeries. Analysis of the ASA score also revealed a strong risk gradient, using ASA 1 as the reference category. Patients classified as ASA 2 had twice the risk of infection (OR = 2.04; 95% CI: 1.76–2.38), while for ASA 3, the risk was almost four times higher (OR = 3.77; 95% CI: 3.20–4.44).

The use of videolaparoscopy was associated with a lower risk of infection, with a rate of 4.1% in video procedures versus 7.7% in conventional surgeries (OR = 1.98; 95% CI: 1.64–2.38; p < 0.0001). The use of implants also proved to be a protective factor, since patients undergoing procedures without implants had an infection rate of 7.9%, while those who used implants had an incidence of 5.5% (OR = 1.47; 95% CI: 1.30–1.66; p < 0.0001).

The analysis of the association by surgical specialty (Table 3) revealed that plastic surgery (OR = 0.44; 95% CI: 0.34–0.57), orthopedics (OR = 0.52; 95% CI: 0.44–0.62), and otorhinolaryngology (OR = 0.53; 95% CI: 0.38–0.72) services presented a significantly lower chance of surgical site infection when compared to patients who underwent procedures in other services. For the urology service, although a trend towards lower risk was observed, the association did not reach statistical significance (OR = 0.87; 95% CI: 0.69–1.09; p = 0.122).

In contrast to the specialties that acted as a protective factor, general surgery was a risk factor for the outcome. Patients in this service had a 25% higher risk of developing infection (OR = 1.25; 95% CI: 1.12–1.38) when compared to the others.

Patients who developed SSI had a longer mean surgery time (3.43 h ± 1.96 h) compared to those who did not had an infection (2.38 h ± 1.54 h). On average, the procedure time for those who had an infection was 1.05 hours longer than for those who did not. The medians differed by 1 h more for surgeries whose patients developed infection (p < 0.0001), suggesting that there is a strong association between the longer time taken to perform the procedure and the presence of postoperative infection (Table 4).

**Table 4:** Association between mean surgery time (in hours) and Surgical Site Infection over 10 years of observation (N = 20778) – Rio de Janeiro, RJ, Brazil, 2023–2024.

	N	Average in hours (±SD)	Min-Max	Median	IQR 25%–75%	p^*^
Infection						
Yes	1491	3.43 (1.96)	0.33–13.98	3.00	2.00–4.50	<0.0001
No	19287	2.38 (1.54)	0.08–23.25	2.00	1.25–3.00	

Notes: IQR = Interquartile Range; *Mann-Whitney U test.

Table 5 presents the results of the multivariate logistic regression analysis, in which the type of surgery and use of implants were not significantly associated with the outcome in the multivariate model. Age, duration of the surgical procedure, procedures that did not use video, potentially contaminated, contaminated and infected, exposure to the general surgery service, and ASA classifications 2 and 3 remained as potential risk factors for infection in this population.

**Table 5:** Multivariate logistic regression analysis of the characteristics related to the presence of Surgical Site Infection over 10 years of observation – Rio de Janeiro, RJ, Brazil, 2023–2024.

	OR	95%CI	*p*
Model 1			
Age (years)	1.01	1.01–1.02	<0.0001
Duration of procedure (hours)	1.34	1.30–1.38	<0.0001
Emergency surgery (*vs.* elective)	1.02	0.83–1.26	0.835
No video use (*vs.* with use of video)	2.73	2.23–3.36	<0.0001
Use of implants	1.05	0.91–1.21	0.537
Potentially contaminated (*vs* clean)	1.50	1.23–1.72	<0.0001
Contaminated (*vs* clean)	2.94	2.49–3.47	<0.0001
Infected (*vs* clean)	2.68	2.00–3.61	<0.0001
General surgery (*vs.* other medical services)	1.67	1.48–1.89	<0.0001
ASA 2 (*vs.* ASA 1)	1.45	1.23–1.72	<0.0001
ASA 3 (*vs.* ASA 1)	1.89	1.56–2.29	<0.0001
Model 2			
Age (years)	1.01	1.00–1.01	<0.0001
Duration of procedure (hours)	1.34	1.30–1.38	<0.0001
No video use (*vs.* with use of video)	2.74	2.23–3.36	<0.0001
Use of implants	1.05	0.91–1.21	0.531
Potentially contaminated (*vs* clean)	1.50	1.30–1.72	<0.0001
Contaminated (*vs* clean)	2.95	2.50–3.58	<0.0001
Infected (*vs* clean)	1.68	1.49–1.89	<0.0001
General surgery (*vs.* other medical services)	1.68	1.49–1.89	<0.0001
ASA 2 (*vs.* ASA 1)	1.45	1.23–1.72	<0.0001
ASA 3 (*vs.* ASA 1)	1.90	1.57–2.29	<0.0001
Model 3			
Age (years)	1.01	1.01–1.02	<0.0001
Duration of procedure (hours)	1.34	1.30–1.38	<0.0001
No video use (*vs.* with use of video)	2.72	2.22–3.34	<0.0001
Potentially contaminated (*vs* clean)	1.52	1.33–1.73	<0.0001
Contaminated (*vs* clean)	3.00	2.57–3.50	<0.0001
Infected (*vs* clean)	2.76	2.10–3.63	<0.0001
General surgery (*vs.* other medical services)	1.68	1.48–1.89	<0.0001
ASA 2 (*vs.* ASA 1)	1.46	1.24–1.72	<0.0001
ASA 3 (*vs.* ASA 1)	1.91	1.58–2.30	<0.0001

Notes: OR = Odds Ratio; CI = Confidence Interval; ASA = American Society of Anesthesiologists.

In the final model (model 3) of the logistic regression, age was associated with a 1% increase in the odds ratio of the event for each additional year (OR = 1.01; 95% CI: 1.01–1.02). The odds ratio observed for the duration of the procedure showed that, for each additional hour of execution, the chance of infection increased by 34% (OR = 1.34; 95% CI: 1.30–1.38). Procedures that did not use video were also associated with a greater chance of infection, with a 172% increase in the event’s odds ratio (OR = 2.72; 95% CI: 2.22–3.34), suggesting that the use of video in surgical procedures may be a potential protective factor for SSI.

The potential for contamination was also investigated and it was observed that surgeries classified as potentially contaminated (RC = 1.52; 95% CI: 1.33–1.73), contaminated (RC = 3.00; 95% CI: 2.57-3.50) and infected (RC = 2.76; 95% CI: 2.10-3.63) were significantly associated with the presence of infection. General surgery, compared with other medical services, showed a 68% increase (OR = 1.68; 95% CI: 1.48–1.89) in the chance of infection. Finally, individuals classified in both ASA 2 (OR = 1.46; 95% CI: 1.24–1.72) and ASA 4 (OR = 1.91; 95% CI: 1.58–2.30) categories had a greater chance of infection when compared to those classified as ASA 1.

## DISCUSSION

In recent decades, there has been a progressive increase in the number of surgical interventions, which intensifies concern about SSIs, whose complications generate both financial and social impacts^9^. This phenomenon is the result of a complex and multifactorial interaction between extrinsic and intrinsic factors, giving each individual a specific risk for developing infections^(12)^.

The identification of risk factors and the construction of a risk predictor model for the occurrence of SSI contributes to the planning and adoption of strategies for prevention and surveillance, which involves a broad approach, with the nurse being one of the main health professionals who must participate rigorously in all phases of surgical care.

The sociodemographic analysis of this study revealed that most procedures were performed on female patients. This result is in line with previous research and may be associated with the fact that women tend to seek health services more frequently, especially for preventive measures and medical follow-up^(14)^.

Still in relation to the variable sex, this research did not find a statistically significant difference in the incidence of surgical site infection between men and women. This result is in line with other findings, such as those from a robust multicenter cohort study of 17,353 patients undergoing gastrointestinal surgery^(15)^, in which, although the incidence of this complication was slightly higher in males, statistical analysis showed no association (p = 0.088). Therefore, the variable sex was not considered to integrate the model proposed here.

Age is a widely recognized risk factor for both impaired wound healing and the development of SSIs. Older patients tend to have a greater number of comorbidities and physiological changes^(16)^, which may lead to greater vulnerability to infection. This scenario was corroborated by a retrospective study with orthopedic patients, in which increasing age stood out as a significant predictor for the occurrence of SSI^(16,17,18,19)^.

Similarly, a meta-analysis that investigated risk factors for postoperative infection in cancer patients showed that the age group over 60 years is significantly associated with a higher risk of SSI^(20)^. Corroborating these findings, the present study also demonstrated a significant association between advancing age and the incidence of SSI, indicating a progressive increase in risk in older age groups. This result highlights the need for rigorous monitoring strategies in older patients undergoing surgical procedures, aiming to minimize infectious complications and optimize clinical outcomes.

The research results highlight that emergency surgery was significantly associated with a higher chance of SSI, demonstrating that emergency surgery increased the chance of SSI by two times when compared to elective procedures, corroborating previous findings in the literature^(19)^. These findings underscore the importance of preventive strategies and intensified surveillance in scenarios where the surgical procedure was performed on an emergency basis. However, after logistic regression, this was not a risk factor to be considered in the modeling proposed in this research.

In a prospective study involving 100 patients from surgical subspecialties, carried out over a period of 6 months, it was observed that SSI rates increased progressively as the ASA classification increased, being especially higher in patients classified as ASA 3 and 4^(19)^, a similar finding identified in this investigation. This result is in line with evidence previously described in the literature and with the SSI risk index^(21,22,23)^ by the CDCs, which had already identified ASA as one of the three main risk factors for SSI, an association that was later corroborated by the predictive model proposed in this study.

Technological advances in surgical techniques, such as the use of video equipment, have revolutionized surgical practice in recent decades and, in this study, as in the literature, demonstrated a protective effect against SSI^(24–25)^. Surgeries classified as contaminated and infected are more likely to develop SSIs when compared to surgeries classified as clean due to their potential for contamination, according to the results of this research and corroborated by previous studies^(15,20)^.

The prolonged duration of the surgical procedure constitutes a significant risk factor for surgical site infection^(26)^. Extended operative time often reflects greater complexity of the procedure, additional technical challenges, and prolonged tissue exposure, elements that, together, create conditions favorable to the development of infectious complications^(16,27–28)^, suggesting that appropriate surgical planning to reduce surgical duration may help reduce the risk of SSI.

The use of implants, in this research, behaved as a protective factor for SSI in the statistical analyses, a finding that conflicts with the available literature^(29)^. Nevertheless, it should be emphasized that this variable did not remain as an independent predictor in the final logistic regression model, suggesting that its apparent protective effect may be associated with other confounding factors not controlled in the analysis.

Still regarding the results above mentioned, it is worth highlighting that the procedures performed by general surgery are significantly associated with a higher risk of SSI when compared to other specialties, being an important predictive factor in the composition of the predictive model. However, despite the internal consistency of the data, comparison with other studies is limited because much of the available evidence aims to build specific predictive models for populations undergoing the same type of procedure. For instance, studies on colorectal surgery often evaluate risks such as bowel preparation and antibiotic prophylaxis, while research addressing other gastrointestinal procedures^(15,30)^ focus on risk factors related to the surgical technique and the patient.

This fragmented approach by procedures generates non-generalizable risk models, hindering their application in general care hospitals, which encompass a diversity of surgical techniques and specialties.

This study has limitations inherent to its retrospective design and the use of secondary data, which may be subject to underreporting and variability in the quality of records. Furthermore, it was conducted with data from a single hospital, which may restrict the generalization of the findings to other institutional settings.

Despite these limitations, the results contribute significantly to the field of nursing and health, by providing robust epidemiological evidence on factors associated with SSI. The identification of independent variables associated with the infectious outcome supports the development of predictive risk models, with potential application in the stratification and prioritization of patients within the scope of active surveillance.

## CONCLUSION

This study establishes a predictive model for the occurrence of surgical site infection based on six variables associated with the outcome studied and easily obtained clinically, such as: age, duration of the procedure, use of video, contamination potential, ASA score, and the general surgery service. The main contribution of this model lies in its wide applicability, as it uses universally measurable parameters. This overcomes a common barrier of more complex models, which often rely on specific laboratory markers and makes comparison between different surgical contexts difficult.

In practice, the model translates into an effective tool for potential risk stratification, allowing healthcare teams to direct preventive strategies more assertively, optimize the use of resources, and, fundamentally, enhance patient safety. The importance of constructing new studies with standardized methodological approaches is also highlighted, aiming to allow more robust cross-sectional analyses and improve risk identification in heterogeneous surgical scenarios.

Thus, the ability to identify profiles of patients at higher risk for SSI represents a significant advance in planning predictive risk management and in surveillance that is more directed to the identified risk. This aspect is particularly relevant in highly complex hospitals, where it can generate substantial impacts on the quality of care and clinical outcomes.

An important limitation of the study is the fact that the model was developed with data from a single hospital center, and emphasizes the need for validation in multiple institutional settings, preferably in hospitals with different complexity profiles and distinct epidemiological characteristics. However, the predictive model proposed in the study brings valuable contributions to improving SSI prevention practices and directing future research on the topic of patient safety.

## Data Availability

The entire dataset supporting the results of this study was published in the article itself.
