# Supplementary material for: Predictive model of surgical infection to enhance patient safety: A retrospective cohort study
Source: Rev Esc Enferm USP. 2025 Dec 1;59:e20250207. doi: 10.1590/1980-220X-REEUSP-2025-0207en (PMC12679714; doi:10.1590/1980-220X-REEUSP-2025-0207en)
Supplement: Translation

# Translation: Modelo preditivo de infecção cirúrgica para fortalecer a segurança do paciente: estudo de coorte retrospectivo

**DOI:** 10.1590/1980-220X-REEUSP-2025-0207pt

**Authors:** Christiany Moçali Gonzalez, Joana de Oliveira Pantoja Freire, Camila Medeiros dos Santos de Cerqueira, Graciele Oroski Paes

**Affiliations:** 1 Universidade Federal do Rio de Janeiro, Escola de Enfermagem Anna Nery, Pós-graduação da Escola de Enfermagem Anna Nery, Rio de Janeiro, RJ, Brasil.

**Keywords:** Medição de Risco, Infecção da Ferida Cirúrgica, Segurança do Paciente

## Abstract

**Objetivo:**

Identificar fatores de risco para infecção do sítio cirúrgico e estabelecer um modelo de predição.

**Método:**

Estudo de coorte retrospectivo com 20.778 cirurgias realizadas entre 2009 e 2019 em hospital universitário do Rio de Janeiro. Foram analisadas variáveis clínicas e operatórias por regressão logística multivariada (p ≤ 0,05).

**Resultados:**

A taxa global de infecção cirúrgica foi de 7,2%. Idade ≥ 41 anos apresentou razão de chance entre 1,52 e 3,77 (p < 0,0001). Cirurgias contaminadas e infectadas aumentaram o risco em três vezes (IC95%: 2,48–3,63). Procedimentos de urgência (RC = 2,04; IC95%: 1,83–2,28) e ASA III (RC = 3,77) foram associados a maior risco. Cada hora adicional de cirurgia aumentou o risco em 34% (RC = 1,34; IC95%: 1,30–1,38). Técnica convencional teve risco 2,7 vezes maior que videolaparoscopia (RC = 2,72; p < 0,0001).

**Conclusão:**

O modelo desenvolvido permite estratificar risco de infecção de sítio cirúrgico com precisão e subsidiar estratégias preventivas, qualificando a vigilância e a gestão do risco cirúrgico em hospitais de alta complexidade.

## INTRODUÇÃO

As infecções do sítio cirúrgico (ISC) são complicações relacionadas aos procedimentos cirúrgicos, com ou sem colocação de implantes, em pacientes internados ou ambulatoriais. Ocorrem quando há pelo menos uma incisão, realizada em um centro cirúrgico, feita através da pele, membrana mucosa ou de uma incisão que foi deixada aberta durante um procedimento cirúrgico anterior^(1)^.

Apresentam uma incidência variável e representam grande desafio na prevenção de infecções relacionadas à assistência à saúde (IRAS), ocupando a terceira posição entre todas as infecções em serviços de saúde e compreendendo, no Brasil, 14% a 16% das IRAS encontradas em pacientes hospitalizados^(2)^.

Em países de alta renda, como os Estados Unidos da América, essa questão também permanece relevante. Dados recentes indicam que as ISC são responsáveis por mais de dois milhões de infecções nosocomiais em pacientes hospitalizados, correspondendo a 20% das principais infecções, evidenciando a gravidade do problema mesmo em sistemas de saúde avançados^(3–4)^. Na Europa, a situação não é diferente, ela é apontada como a segunda infecção mais recorrente, sendo responsável por cerca de 500.000 casos por ano^(5)^.

Aumento do tempo de internação, em até três vezes^(5–6)^, elevação dos custos hospitalares e o risco de morte de 2 a 11 vezes maior em comparação com pacientes que não desenvolvem ISC^(3,7–8)^ são impactos significativos que não apenas sobrecarregam os sistemas hospitalares, mas também aumentam os custos operacionais, limitam a disponibilidade de leitos para outros pacientes e exigem atenção prioritária no contexto da assistência à saúde^(9–10)^.

Esses fatores não afetam somente os hospitais, mas também os próprios pacientes, que enfrentam maior sofrimento, complicações no pós-operatório e impactos socioeconômicos, como afastamento prolongado do trabalho^(7)^. À luz desses fatos, a atuação de equipe multiprofissional é crucial para a realização de estratégias rigorosas de vigilância, controle de infecções e aprimoramento das práticas cirúrgicas, sendo, portanto, essenciais para mitigar esses riscos e garantir melhores desfechos clínicos^(11)^.

Diante dos impactos apresentados e considerando a sua evitabilidade, torna-se imprescindível a implementação de medidas de prevenção para mitigar os riscos de ocorrência desse evento nas instituições de saúde, conforme orientam as diretrizes internacionais da Organização Mundial da Saúde (OMS) e as nacionais da Agência de Vigilância Sanitária (ANVISA).

A implementação dessas estratégias reflete um compromisso global com a segurança do paciente e a melhoria contínua dos cuidados cirúrgicos, uma vez que essas medidas visam não apenas reduzir as taxas de ISC, mas também minimizar os riscos de complicações graves, garantindo a qualidade da assistência e promovendo procedimentos cirúrgicos mais seguros.

Neste contexto, o enfermeiro tem papel de destaque, uma vez que este profissional é responsável pelo planejamento e implementação dos cuidados de enfermagem necessários e pela educação em saúde. Além disso, deve ter conhecimento sobre as complicações, direcionado para a prevenção e detecção precoce destas, auxiliando efetivamente no bem-estar do paciente e na nova condição de vida^(12)^.

Assim, é imperativo que tenhamos um conhecimento sólido sobre a relação dos fatores de risco com o desfecho da ISC, uma vez que esses fatores são vistos como substitutos da causa subjacente e utilizados para prever desfechos. Quando um fator se mostra repetidamente associado às ISC, ele se torna um indicativo mais confiável para o desenvolvimento de estratégias de prevenção e vigilância. Além disso, fatores de risco consistentes podem ser utilizados para padronizar as taxas de ISC entre pacientes, permitindo comparações mais precisas e contribuindo para iniciativas de melhoria da qualidade^(7)^.

Frente ao exposto, estudos com análise abrangente de uma grande coorte de pacientes, juntamente com a identificação de fatores de risco e proteção, aumentam nossa compreensão dessa complexa complicação cirúrgica. Entender e estratificar esse risco é essencial para melhorar desfechos, orientar decisões de vigilância e alocar recursos de forma eficiente, uma vez que essas taxas em si são difíceis de interpretar sem estratificação de risco^(7,13)^. Portanto, uma ferramenta de estratificação de risco é um elemento-chave no caminho diagnóstico e permite identificar, de forma antecipada, indivíduos que apresentam fatores de risco elevados, possibilitando a implementação de estratégias de monitoramento e prevenção mais eficazes, objetivando melhoria contínua na qualidade e segurança do paciente.

Neste contexto, esse estudo tem como objetivo identificar os fatores de risco de infecção do sítio cirúrgico em cirurgias realizadas em um hospital universitário e estabelecer um modelo de predição.

## MÉTODO

### Tipo de Estudo

Trata-se de uma pesquisa de abordagem quantitativa, observacional do tipo coorte retrospectiva.

### Local de Coleta de Dados

O local escolhido para desenvolver o estudo foi o Hospital Universitário Federal, referência no tratamento de diversas patologias de alta complexidade e um importante centro de assistência, ensino, pesquisa e extensão, localizado no Rio de Janeiro.

### Amostra do Estudo

A amostra utilizada na pesquisa foi extraída do banco de dados da vigilância cirúrgica, pertencente à Coordenação de Controle de Infecção Hospitalar (CCIH), interligado ao sistema de prontuário eletrônico do hospital, compreendendo o período de 2009 a 2019. O recorte temporal adotado nesta pesquisa foi definido visando garantir a consistência e a comparabilidade dos dados. Optou-se pela exclusão dos anos de 2020 e 2021 em virtude do impacto direto da pandemia de COVID-19 sobre os fluxos cirúrgicos e a dinâmica hospitalar. Durante esse período, houve uma redução significativa na realização de procedimentos eletivos, priorização de cirurgias de urgência e emergência, além de reestruturações nos serviços de saúde voltadas ao enfrentamento da crise sanitária.

Essas alterações comprometeram a regularidade das práticas cirúrgicas e dos processos de notificação e vigilância de infecções relacionadas à assistência à saúde, especialmente no que se refere à ISC. Como consequência, os dados desses anos apresentam características atípicas, que poderiam introduzir vieses na análise estatística e prejudicar a identificação de padrões epidemiológicos e fatores de risco em contextos assistenciais estáveis. A pesquisa incluiu 20.778 cirurgias.

### Critérios de Seleção

Foram incluídos no estudo os dados das cirurgias que atenderam aos critérios estabelecidos pela Agência Nacional de Vigilância Sanitária (ANVISA): aquelas cirurgias cuja data de alta hospitalar do paciente diferisse da data do procedimento e aquelas nas quais os pacientes possuíam idade igual ou superior a 18 anos. Foram excluídas as reoperações realizadas em um intervalo inferior a 30 dias após a primeira intervenção, cirurgias realizadas em hemodinâmica e procedimentos cirúrgicos indicados para o tratamento de trauma agudo.

### Coleta de Dados

A coleta dos dados ocorreu no período de outubro de 2023 a abril de 2024.

### Análise dos Dados

Analisou-se a infecção de sítio cirúrgico nos procedimentos cirúrgicos e seus possíveis fatores de risco. A categorização da ISC baseou-se na ausência e na presença da infecção, podendo essa ser incisional, superficial ou profunda, de órgãos e espaços.

Como variáveis independentes foram considerados os fatores de risco à ISC, aqueles cuja presença está associada à maior probabilidade de que uma infecção venha a se desenvolver: sexo; potencial de contaminação da ferida cirúrgica (limpa, potencialmente contaminada, contaminada e infectada); condições clínicas do paciente no pré-operatório, analisado pelo ASA I, II, III, IV e V, critério proposto pela *American Society of Anesthesiologists*; emergência (não e sim); implante (não e sim); tipo de procedimento cirúrgico; e duração da cirurgia.

As análises foram realizadas por meio de estatística descritiva, com cálculo de média, mediana e desvio padrão (DP) para variáveis quantitativas. Já as variáveis qualitativas foram descritas com base na frequência absoluta (n) e relativa (%). As análises bivariadas que incluíram variáveis qualitativas ou categóricas compreenderam o teste qui-quadrado e a razão de chance.

Para as análises bivariadas, a normalidade das variáveis quantitativas ou contínuas foi primeiramente avaliada pelo teste de Shapiro-Wilk. Uma vez que os dados não apresentaram distribuição normal, utilizou-se o teste não paramétrico U de Mann-Whitney para verificar a associação entre as variáveis demográficas e clínicas e o desfecho.

Na análise de regressão logística multivariada foi utilizado o método *backward*, isto é, a cada passo foram retiradas do modelo as variáveis que não se associaram significativamente ao desfecho. Assim, ao final do modelo multivariado, permaneceram aquelas variáveis significativamente associadas à presença de infecção pós-cirúrgica.

De forma semelhante foi verificada, por meio de análises bivariadas, a associação entre as variáveis demográficas e clínicas e a infecção hospitalar. Assumiu-se para as análises o nível de significância de 5% (p ≤ 0,05) e intervalo de confiança de 95%. Todas as análises foram elaboradas no programa IBM SPSS (v.16.0) e foi usado o programa Microsoft Excel para elaboração das figuras.

### Aspectos Éticos

O estudo obteve aprovação do Comitê de Ética em Pesquisa (CEP), sob o parecer de nº 6.231.690, atendendo à Resolução nº 466/2012, do Conselho Nacional de Saúde (CNS). Por se tratar de um estudo retrospectivo realizado a partir de fontes secundárias inerentes ao processo de vigilância e controle de IRAS já realizado pela CCIH do local de estudo, foi concedida a dispensa da assinatura do Termo de Consentimento Livre e Esclarecido para realizar o estudo.

## RESULTADOS

O estudo incluiu a análise de 20.778 cirurgias eletivas e de urgência, distribuídas ao longo de 10 anos de contabilização. Observou-se que os anos de 2013 (n = 2119; 10,2%) e 2016 (n = 2106; 10,1%) foram os que concentraram a maior prevalência de cirurgias realizadas.

A maioria dos procedimentos ocorreu com pacientes do sexo feminino (58,8%). A idade dos pacientes variou de 18 a 97 anos, com média de 52,0 anos (dp = 16,3 anos) e maior prevalência de eventos cirúrgicos (23,0%) para a faixa etária de 51 a 60 anos (Tabela 1).

**Tabela 1:** Análises descritivas das variáveis demográficas e clínicas de pacientes submetidos a procedimentos cirúrgicos entre os anos de 2009 e 2019 (N = 20778) – Rio de Janeiro, RJ, Brasil, 2023–2024.

Variáveis	N	%	Min-Máx	Média (±DP)
Sexo				
Masculino	8622	41,5		
Feminino	12156	58,5		
Idade			18–97	52,0 (±16,3)
Faixa etária				
De 18 a 30 anos	2551	12,3		
31 a 40 anos	2746	13,2		
41 a 50 anos	3819	18,4		
51 a 60 anos	4875	23,5		
61 a 70 anos	4017	19,3		
71 ou mais	2770	13,3		
Duração em horas			0,083–23,3	2,5 (±1,6)
ASA				
1	6273	30,2		
2	10375	49,9		
3	3818	18,4		
4	305	1,5		
5	6	0,0		
Tipo de cirurgia				
Eletiva	19390	93,3		
Urgência	1388	6,7		
Desfecho				
Óbito	634	3,1		
Alta	20144	96,9		
Uso de vídeo				
Não	17607	84,7		
Sim	3171	15,3		
Utilizou implantes				
Não	15714	75,6		
Sim	5064	24,4		

Notas: DP = desvio padrão; ASA = *American Society of Anesthesiologists*.

Nos homens, a idade média foi de 53,03 (dp = 16,90) e nas mulheres foi de 51,29 (dp = 15,78). A análise de variância para o sexo em relação às idades, pela técnica de Mann-Whitney, apresentou violação da hipótese nula (U = 5,62 × 10^7^; p < 0,001; VS-MPR = 8,51 × 10^15^; rbs = 0,07), indicando padrões distintos de variação da idade entre os sexos.

A contabilização do tempo de duração das cirurgias realizadas mostrou que, em média, foram dedicadas 2,46 horas (±1,60). A maioria dos procedimentos foi de caráter eletivo (93,3%) e não necessitou de reoperação (97,6%), enquanto 6,7% foram cirurgias de urgência. Outra característica observada foi o ASA, cuja classificação mais frequentemente observada foi a 2 (49,9%). A análise dos desfechos clínicos revelou que 96,9% (n = 20.144) dos pacientes receberam alta hospitalar, enquanto 3,1% (n = 634) evoluíram para óbito. (Tabela 1)

O uso de implantes foi registrado em 24,4% (n = 5.064) dos procedimentos, reforçando a importância desses dispositivos no manejo cirúrgico, enquanto 15,3% (n = 3.171) das cirurgias utilizaram videocirurgia.

Na amostra do estudo, registraram-se 1.491 ocorrências de infecção de sítio cirúrgico, perfazendo uma taxa de incidência de ISC de aproximadamente 7,2%. Destas, a maioria (54,59%) foi superficial e 31,52% foram intracavitárias. O local de identificação da infecção foi, em grande parte, nos ambulatórios de egressos (54,19%), conforme disposto na Tabela 2.

**Tabela 2:** Caracterização da ocorrência de Infecção de Sítio Cirúrgico nos participantes ao longo de 10 anos de observação (N = 20.778) – Rio de Janeiro, RJ, Brasil, 2023–2024.

Variáveis	N	%
Ocorrência de infecção		
Sim	1.491	7,18
Não	19.287	92,82
Tipo de infecção		
Superficial	814	54,59
Intracavitária	470	31,52
Profunda	207	13,88
Locais de diagnóstico da infecção		
Ambulatório	808	54,19
Enfermaria	683	45,81

Em relação ao sexo, não houve diferença estatisticamente significativa na incidência de infecção entre homens (7,0%) e mulheres (7,4%) (RC = 1,05; IC95%: 0,94–1,17; p = 0,374). A avaliação da faixa etária, por sua vez, revelou uma associação significativa com a presença de infecção (p < 0,0001). Observou-se que, com o avanço da idade, a chance do desfecho torna-se maior nos grupos mais velhos (a partir de 41–50 anos) quando comparados aos mais jovens (18–30 anos), conforme demonstrado na Tabela 3.

**Tabela 3:** Tipos de serviços cirúrgicos e fatores associados à Infecção de Sítio Cirúrgico ao longo de 10 anos de observação (N = 20778) – Rio de Janeiro, RJ, Brasil, 2023–2024.

Características estudadas	Presença de infecção				RC (IC95%)	p
	Não		Sim			
	n	%	n	%		
Sexo						
Masculino	11300	93,0	856	7,0	1,05 (0,94–1,17)	0,374
Feminino	7987	92,6	635	7,4		
Faixa etária						
De 18 a 30 anos	2442	95,7	109	4,3	1,0	
31 a 40 anos	2616	95,3	130	4,7	1,11 (0,86–1,45)	0,419
41 a 50 anos	3576	93,6	243	6,4	1,52 (1,21–1,92)	< 0,0001
51 a 60 anos	4518	92,7	357	7,3	1,77 (1,42–2,21)	< 0,0001
61 a 70 anos	3655	91,0	362	9,0	2,22 (1,78–2,77)	< 0,0001
71 ou mais	2480	89,5	290	10,5	2,62 (2,09–3,29)	< 0,0001
Tipo de cirurgia						
Urgência	1209	87,1	179	12,9	2,04 (1,73–2,41)	< 0,0001
Eletiva	18078	93,2	1312	6,8	1,0	
ASA						
Classificação 1	6045	96,4	2299	3,6	1,0	
Classificação 2	9629	92,8	746	7,2	2,04 (1,76–2,38)	< 0,0001
Classificação 3	3340	87,5	478	12,5	3,77 (3,20–4,44)	< 0,0001
Potencial de contaminação						
Limpa	11130	94,7	626	5,3	1,0	
Potencialmente contaminada	5955	92,9	457	7,1	1,37 (1,21–1,55)	< 0,0001
Contaminada	1726	84,2	323	15,8	3,33 (2,88–3,84)	< 0,0001
Infectada	476	84,8	85	15,2	3,18 (2,49–4,01)	< 0,0001
Uso de vídeo						
Não	16245	92,3	1362	7,7	1,98 (1,64–2,38)	< 0,0001
Sim	3042	95,9	129	4,1	1,0	
Uso de implantes						
Não	13379	92,1	1146	7,9	1,47 (1,30–1,66)	< 0,0001
Sim	5908	94,5	345	5,5	1,0	
Otorrinolaringologia						
Sim	1017	95,9	43	4,1	0,53 (0,38–0,72)	< 0,0001
Não	18270	92,7	1448	7,3	1,0	
Neurocirurgia						
Sim	1215	92,5	99	7,5	1,06 (0,86–1,31)	0,603
Não	18072	92,8	1392	7,2	1,0	
Urologia						
Sim	1255	93,7	85	6,3	0,87 (0,69–1,09)	0,122
Não	18032	92,8	1406	7,2	1,0	
Ginecologia						
Sim	1507	92,1	129	7,9	1,12 (0,93–1,35)	0,247
Não	17780	92,9	1362	7,1	1,0	
Cirurgia Plástica						
Sim	1684	96,6	60	3,4	0,44 (0,34–0,57)	< 0,0001
Não	17603	92,5	1431	7,5	1,0	
Ortopedia e trauma						
Sim	3329	95,8	147	4,2	0,52 (0,44–0,62)	< 0,0001
Não	15958	92,2	1344	7,8	1,0	
Cirurgia Geral						
Sim	7246	91,9	638	8,1	1,25 (1,12–1,38)	< 0,0001
Não	12041	93,4	853	6,6	1,0	

Notas: RC = Razão de Chance; IC = Intervalo de Confiança; ASA = *American Society of Anesthesiologists*.

A comparação de pacientes na faixa de 31 a 40 anos com o grupo mais jovem não revelou associação significativa com o desfecho (RC = 1,11; IC95%:0,86–1,45). No entanto, para a faixa etária seguinte, o risco de ISC foi 1,52 vezes maior em relação ao mesmo grupo de referência (IC95%:1,21–1,92). Em síntese, observou-se aumento na chance de infecção com o aumento da faixa etária.

O potencial de contaminação da cirurgia foi avaliado por quatro categorias, utilizando-se a classificação de cirurgia limpa como referência. Em comparação a ela, observou-se que pacientes submetidos a procedimentos potencialmente contaminados apresentaram uma chance 37% maior de desenvolver infecção. Para os procedimentos contaminados, o risco foi 3,33 vezes maior (IC95%: 2,88–3,84). Já nos procedimentos infectados, a chance de desenvolver a complicação foi 3,18 vezes superior (IC95%: 2,49–4,01).

Pessoas expostas a cirurgias de urgência tiveram 2,04 vezes a chance (IC95%: 1,73–2,41) de apresentarem infecção quando comparadas àquelas submetidas a cirurgias eletivas. A análise do escore ASA também revelou um forte gradiente de risco, utilizando ASA 1 como categoria de referência. Pacientes classificados como ASA 2 tiveram o dobro do risco de infecção (RC = 2,04; IC95%: 1,76–2,38), enquanto para ASA 3, o risco foi quase quatro vezes maior (RC = 3,77; IC95%: 3,20–4,44).

A utilização de videolaparoscopia foi associada a um menor risco de infecção, com taxa de 4,1% nos procedimentos por vídeo contra 7,7% nas cirurgias convencionais (RC = 1,98; IC95%: 1,64–2,38; p < 0,0001). O uso de implantes também se mostrou um fator protetor, uma vez que pacientes submetidos a procedimentos sem implantes apresentaram uma taxa de infecção de 7,9%, enquanto aqueles que utilizaram implantes tiveram uma incidência de 5,5% (RC = 1,47; IC95%: 1,30–1,66; p < 0,0001).

A análise da associação por especialidade cirúrgica (Tabela 3) revelou que os serviços de cirurgia plástica (RC = 0,44; IC95%: 0,34–0,57), ortopedia (RC = 0,52; IC95%: 0,44–0,62) e otorrinolaringologia (RC = 0,53; IC95%: 0,38–0,72) apresentaram uma chance significativamente menor de infecção de sítio cirúrgico quando comparados aos pacientes que realizaram procedimentos em outros serviços. Para o serviço de urologia, embora tenha sido observada uma tendência de risco menor, a associação não alcançou significância estatística (RC = 0,87; IC95%: 0,69–1,09; p = 0,122).

Em contraste com as especialidades que atuaram como fator de proteção, a cirurgia geral apresentou-se como um fator de risco para o desfecho. Pacientes deste serviço tiveram um risco 25% superior de desenvolver infecção (RC = 1,25; IC95%: 1,12–1,38) quando comparados aos demais.

Os pacientes que desenvolveram ISC tiveram tempo médio de cirurgia maior (3,43 h±1,96 h) em comparação com aqueles que não desenvolveram infecção (2,38 h±1,54 h). Em média, o tempo de duração do procedimento daqueles que apresentaram infecção teve, em média, 1,05 h a mais quando comparados aos que não tiveram infecção. As medianas diferiram em 1 h a mais para as cirurgias cujos pacientes desenvolveram infecção (p < 0,0001), sugerindo haver forte associação entre o maior tempo de realização do procedimento e a presença de infecção pós-operatória (Tabela 4).

**Tabela 4:** Associação entre o tempo médio (em horas) de cirurgia e a Infecção de Sítio Cirúrgico ao longo de 10 anos de observação (N = 20778) – Rio de Janeiro, RJ, Brasil, 2023–2024.

	N	Média em horas (±DP)	Min-Max	Mediana	IIQ 25%–75%	p^*^
Infecção						
Sim	1491	3,43 (1,96)	0,33–13,98	3,00	2,00–4,50	< 0,0001
Não	19287	2,38 (1,54)	0,08–23,25	2,00	1,25–3,00	

Notas: IIQ = Intervalo Interquartil; *teste U de Mann-Whitney.

A Tabela 5 apresenta os resultados da análise de regressão logística multivariada, na qual não se associaram significativamente ao desfecho no modelo multivariado o tipo de cirurgia e uso de implantes. Permaneceram como potenciais fatores de risco para infecção nesta população a idade, a duração do procedimento cirúrgico, procedimentos que não usaram vídeo, potencialmente contaminada, contaminada e infectada, exposição ao serviço de cirurgia geral e as classificações ASA 2 e 3.

**Tabela 5:** Análise de regressão logística multivariada entre as características relacionadas à presença de Infecção de Sítio Cirúrgico ao longo de 10 anos de observação – Rio de Janeiro, RJ, Brasil, 2023–2024.

	RC	IC95%	*p*
Modelo 1			
Idade (anos)	1,01	1,01–1,02	< 0,0001
Duração do procedimento (horas)	1,34	1,30–1,38	< 0,0001
Cirurgia de urgência (*vs.* eletiva)	1,02	0,83–1,26	0,835
Sem uso de vídeo (*vs.* com uso de vídeo)	2,73	2,23–3,36	< 0,0001
Uso de implantes	1,05	0,91–1,21	0,537
Potencialmente contaminada (*vs* limpa)	1,50	1,23–1,72	< 0,0001
Contaminada (*vs* limpa)	2,94	2,49–3,47	< 0,0001
Infectada (*vs* limpa)	2,68	2,00–3,61	< 0,0001
Cirurgia geral (*vs.* demais serviços médicos)	1,67	1,48–1,89	< 0,0001
ASA 2 (*vs.* ASA 1)	1,45	1,23–1,72	< 0,0001
ASA 3 (*vs.* ASA 1)	1,89	1,56–2,29	< 0,0001
Modelo 2			
Idade (anos)	1,01	1,00–1,01	< 0,0001
Duração do procedimento (horas)	1,34	1,30–1,38	< 0,0001
Sem uso de vídeo (*vs.* com uso de vídeo)	2,74	2,23–3,36	< 0,0001
Uso de implantes	1,05	0,91–1,21	0,531
Potencialmente contaminada (*vs* limpa)	1,50	1,30–1,72	< 0,0001
Contaminada (*vs* limpa)	2,95	2,50–3,58	< 0,0001
Infectada (*vs* limpa)	1,68	1,49–1,89	< 0,0001
Cirurgia geral (*vs.* demais serviços médicos)	1,68	1,49–1,89	< 0,0001
ASA 2 (*vs.* ASA 1)	1,45	1,23–1,72	< 0,0001
ASA 3 (*vs.* ASA 1)	1,90	1,57–2,29	< 0,0001
Modelo 3			
Idade (anos)	1,01	1,01–1,02	< 0,0001
Duração do procedimento (horas)	1,34	1,30–1,38	< 0,0001
Sem uso de vídeo (*vs.* com uso de vídeo)	2,72	2,22–3,34	< 0,0001
Potencialmente contaminada (*vs* limpa)	1,52	1,33–1,73	< 0,0001
Contaminada (*vs* limpa)	3,00	2,57–3,50	< 0,0001
Infectada (*vs* limpa)	2,76	2,10–3,63	< 0,0001
Cirurgia geral (*vs.* demais serviços médicos)	1,68	1,48–1,89	< 0,0001
ASA 2 (*vs.* ASA 1)	1,46	1,24–1,72	< 0,0001
ASA 3 (*vs.* ASA 1)	1,91	1,58–2,30	< 0,0001

Notas: RC = Razão de Chance; IC = Intervalo de Confiança; ASA = *American Society of Anesthesiologists*; *vs*. = *versus*.

No modelo final (modelo 3) da regressão logística, a idade associou-se a um aumento de 1% na razão de chance do evento para cada ano adicional (RC = 1,01; IC95%: 1,01–1,02). Já a razão de chance observada para o tempo de duração do procedimento evidenciou que, a cada hora adicional de execução aumentava-se em 34% a chance de infecção (RC = 1,34; IC95%:1,30–1,38). Procedimentos que não usaram vídeo também se associaram à maior chance de infecção, com um incremento de 172% na razão de chance do evento (RC = 2,72; IC95%: 2,22–3,34), sugerindo que o uso de vídeo em procedimentos cirúrgicos pode ser um potencial fator de proteção para a ISC.

Também foi investigado o potencial de contaminação e observou-se que cirurgias classificadas como potencialmente contaminadas (RC = 1,52; IC95%: 1,33–1,73), contaminadas (RC = 3,00; IC95%: 2,57–3,50) e infectadas (RC = 2,76; IC95%: 2,10–3,63) mostraram-se significativamente associadas à presença de infecção. A cirurgia geral, em comparação com os demais serviços médicos, mostrou aumento de 68% (RC = 1,68; IC95%: 1,48–1,89) na chance de infecção. Por fim, indivíduos classificados tanto na categoria ASA 2 (RC = 1,46; IC95%: 1,24–1,72) quanto ASA 4 (RC = 1,91; IC95%: 1,58–2,30) apresentaram maior chance de infecção quando comparados aos classificados como ASA 1.

## DISCUSSÃO

Nas últimas décadas, tem-se observado um aumento progressivo no número de intervenções cirúrgicas, o que intensifica a preocupação com as ISC, cujas complicações geram impactos tanto financeiros quanto sociais^9^. Esse fenômeno é resultado de uma interação complexa e multifatorial entre fatores extrínsecos e intrínsecos, conferindo a cada indivíduo um risco específico para o desenvolvimento de infecções^(12)^.

A identificação dos fatores de riscos e a construção de um modelo preditor de risco para ocorrência da ISC contribui no planejamento e adoção de estratégias para prevenção e vigilância, o que envolve abordagem ampla, sendo o enfermeiro um dos principais profissionais da saúde que deve participar com rigor em todas as fases do cuidado da linha cirúrgica.

A análise sociodemográfica deste estudo revelou que a maioria dos procedimentos foi realizada em pacientes do sexo feminino. Esse resultado está em consonância com pesquisas anteriores e pode estar associado ao fato de que as mulheres tendem a procurar mais os serviços de saúde, especialmente para medidas preventivas e de acompanhamento médico^(14)^.

Ainda em relação à variável sexo, esta pesquisa não encontrou diferença estatisticamente significativa na incidência de infecção de sítio cirúrgico entre homens e mulheres. Este resultado está em consonância com outros achados, como os de um robusto estudo de coorte multicêntrico com 17.353 pacientes submetidos à cirurgia gastrointestinal^(15)^, no qual, embora a incidência dessa complicação tenha sido ligeiramente maior no sexo masculino, a análise estatística não demonstrou associação (p = 0,088). Dessa forma, a variável sexo não foi considerada para integrar o modelo aqui proposto.

A idade é um fator de risco amplamente reconhecido tanto para o comprometimento da cicatrização de feridas quanto para o desenvolvimento de infecções do sítio cirúrgico (ISC). Pacientes mais idosos tendem a apresentar um número maior de comorbidades e alterações fisiológicas^(16)^, o que pode inclinar a uma maior vulnerabilidade à infecção. Esse cenário foi corroborado por um estudo retrospectivo com pacientes ortopédicos, no qual o aumento da idade se destacou como um preditor significativo para a ocorrência de ISC^(16–19)^.

De forma semelhante, uma metanálise que investigou fatores de risco para infecção pós-operatória em pacientes oncológicos evidenciou que a faixa etária acima de 60 anos está significativamente associada a um maior risco de ISC^(20)^. Corroborando esses achados, o presente estudo também demonstrou uma associação significativa entre o avanço da idade e a incidência de ISC, indicando um aumento progressivo do risco em grupos etários mais elevados. Esse resultado ressalta a necessidade de estratégias de um monitoramento rigoroso em pacientes idosos submetidos a procedimentos cirúrgicos, visando minimizar complicações infecciosas e otimizar os desfechos clínicos.

Os resultados da pesquisa ressaltam que a cirurgia de emergência esteve significativamente associada a uma maior chance de infecção do sítio cirúrgico (ISC), demonstrando que a cirurgia de emergência aumentou em duas vezes a chance de ISC quando comparada a procedimentos eletivos, corroborando achados prévios na literatura^(19)^. Tais achados advertem para a importância de estratégias preventivas e de vigilância intensificadas em cenários onde o procedimento cirúrgico tenha sido realizado em caráter emergencial. No entanto, após a realização da regressão logística, este não foi um fator de risco a ser considerado na modelagem proposta nessa pesquisa.

Em um estudo prospectivo envolvendo 100 pacientes de subespecialidades cirúrgicas, realizado durante o período de 6 meses, observou-se que as taxas de ISC aumentavam progressivamente conforme a classificação ASA se elevava, sendo especialmente mais altas em pacientes classificados como ASA 3 e 4^(19)^, achado semelhante identificado nesta investigação. Esse resultado está alinhado com evidências previamente descritas na literatura e com o índice de risco de ISC do CDC^(21–23)^, que já havia apontado a ASA como um dos três principais fatores de risco para ISC, associação que foi posteriormente corroborada pelo modelo preditivo proposto neste estudo.

Os avanços tecnológicos nas técnicas cirúrgicas, como a utilização de equipamento de vídeo, revolucionaram a prática cirúrgica nas últimas décadas e, nesse estudo, como na literatura, demonstraram efeito protetor contra ISC^(24–25)^. Cirurgias classificadas como contaminadas e infectadas têm mais chances de evoluírem com ISC quando comparadas com as cirurgias classificadas como limpas pelo potencial de contaminação, conforme os resultados dessa pesquisa e corroborada por estudos anteriores^(15,20)^.

A duração prolongada do ato cirúrgico constitui um fator de risco significativo para infecção do sítio cirúrgico^(26)^. O tempo operatório estendido frequentemente reflete maior complexidade do procedimento, desafios técnicos adicionais e exposição tecidual prolongada, elementos que, em conjunto, criam condições favoráveis ao desenvolvimento de complicações infecciosas^(16,27–28)^, sugerindo que um planejamento cirúrgico apropriado para reduzir a duração cirúrgica pode ajudar a reduzir o risco de ISC.

O uso de implantes, nessa pesquisa, comportou-se como um fator protetor para ISC nas análises estatísticas, um achado que vai de encontro à literatura disponível^(29)^. Entretanto, é importante destacar que essa variável não se manteve como preditor independente no modelo final de regressão logística, podendo ser sugerido que seu aparente efeito protetor pode estar associado a outros fatores de confusão não controlados na análise.

Ainda sobre os resultados aqui em tela, destaca-se que os procedimentos realizados pela cirurgia geral estão significativamente associados a um maior risco de ISC quando comparados a outras especialidades, sendo um fator preditivo importante na composição do modelo preditor. No entanto, apesar da consistência interna dos dados, a comparação com outros estudos é limitada porque grande parte das evidências disponíveis tem como objetivo a construção de modelos preditivos específicos para populações submetidas ao mesmo tipo de procedimento. Por exemplo, estudos sobre cirurgia colorretal frequentemente avaliam riscos como preparo intestinal e antibioticoprofilaxia, enquanto pesquisas que abordam outros procedimentos gastrointestinais^(15,30)^ focam em fatores de risco relacionados à técnica cirúrgica e ao paciente.

Essa abordagem fragmentada por procedimentos gera modelos de risco não generalizáveis, dificultando sua aplicação em hospitais de atendimento generalista, que engloba uma diversidade de técnicas e especialidades cirúrgicas.

Este estudo apresenta limitações inerentes ao delineamento retrospectivo e ao uso de dados secundários, que podem estar sujeitos à subnotificação e variabilidade na qualidade dos registros. Além disso, foi conduzido com dados de um único hospital, o que pode restringir a generalização dos achados para outras realidades institucionais.

Apesar dessas limitações, os resultados contribuem significativamente para a área da enfermagem e da saúde, ao oferecer evidências epidemiológicas robustas sobre fatores associados à ISC. A identificação de variáveis independentes associadas ao desfecho infeccioso subsidia o desenvolvimento de modelos preditivos de risco, com potencial aplicação na estratificação e priorização de pacientes no âmbito da vigilância ativa.

## CONCLUSÃO

Este estudo estabelece um modelo preditivo para a ocorrência de infecção de sítio cirúrgico baseado em seis variáveis com associação ao desfecho estudado e de fácil obtenção clínica, tais como: idade, duração do procedimento, uso de vídeo, potencial de contaminação, escore ASA e o serviço de cirurgia geral. A principal contribuição deste modelo reside em sua ampla aplicabilidade, visto que utiliza parâmetros universalmente mensuráveis. Isso supera uma barreira comum de modelos mais complexos, que frequentemente dependem de marcadores laboratoriais específicos e dificultam a comparação entre diferentes contextos cirúrgicos.

Na prática, o modelo traduz-se em uma ferramenta eficaz para uma potencial estratificação de risco, permitindo que as equipes de saúde direcionem estratégias preventivas de forma mais assertiva, otimizem o uso de recursos e, fundamentalmente, fortaleçam a segurança do paciente. Ressalta-se, também, a importância construção de novos estudos com abordagens metodológicas padronizadas, visando permitir análises transversais mais robustas e aprimorar a identificação de risco em cenários cirúrgicos heterogêneos.

Assim, a capacidade de identificar perfis de pacientes com maior risco para ISC representa um avanço significativo no planejamento de uma gestão de risco preditivo e em uma vigilância mais direcionada ao risco identificado. Este aspecto é particularmente relevante em hospitais de alta complexidade, onde pode gerar impactos substanciais na qualidade da assistência e nos desfechos clínicos.

Ressalta-se como limitação importante do estudo o fato de o modelo ter sido desenvolvido com dados de um único centro hospitalar, e enfatiza a necessidade de validação em múltiplos cenários institucionais, preferencialmente em hospitais com diferentes perfis de complexidade e características epidemiológicas distintas. No entanto, o modelo preditivo proposto no estudo traz contribuições valiosas para o aprimoramento das práticas de prevenção de ISC e para o direcionamento de pesquisas futuras na temática da segurança do paciente.

## Data Availability

Todo o conjunto de dados que dá suporte aos resultados deste estudo foi publicado no próprio artigo.

# Translation: Modelo predictivo de infección quirúrgica para fortalecer la seguridad del paciente: estudio de cohorte retrospective

**DOI:** 10.1590/1980-220X-REEUSP-2025-0207es

**Authors:** Christiany Moçali Gonzalez, Joana de Oliveira Pantoja Freire, Camila Medeiros dos Santos de Cerqueira, Graciele Oroski Paes

**Affiliations:** 1 Universidade Federal do Rio de Janeiro, Escola de Enfermagem Anna Nery, Pós-graduação da Escola de Enfermagem Anna Nery, Rio de Janeiro, RJ, Brasil.

**Keywords:** Medición de Riesgo, Infección de la Herida Quirúrgica, Seguridad del Paciente

## Abstract

**Objetivo:**

Identificar los factores de riesgo de infección del sitio quirúrgico y establecer un modelo de predicción.

**Método:**

Estudio de cohorte retrospectivo con 20.778 cirugías realizadas entre 2009 y 2019 en un hospital universitario de Río de Janeiro. Las variables clínicas y operatorias fueron analizadas mediante regresión logística multivariada (p ≤ 0,05).

**Resultados:**

La tasa global de infección quirúrgica fue del 7,2%. La edad ≥41 años presentó un odds ratio entre 1,52 y 3,77 (p < 0,0001). Las cirugías contaminadas e infectadas triplicaron el riesgo (IC del 95%: 2,48–3,63). Los procedimientos urgentes (OR = 2,04; IC 95%: 1,83–2,28) y ASA III (OR = 3,77) se asociaron con un mayor riesgo. Cada hora adicional de cirugía aumentó el riesgo en un 34% (OR = 1,34; IC 95%: 1,30–1,38). La técnica convencional tuvo un riesgo 2,7 veces mayor que la videolaparoscopia (RC = 2,72; p < 0,0001).

**Conclusión:**

El modelo desarrollado permite una estratificación precisa del riesgo de infección del sitio quirúrgico y apoya estrategias preventivas, mejorando la vigilancia y la gestión del riesgo quirúrgico en hospitales de alta complejidad.

## INTRODUCCIÓN

Las infecciones del sitio quirúrgico (ISQ) son complicaciones relacionadas con procedimientos quirúrgicos, con o sin colocación de implantes, en pacientes hospitalizados o ambulatorios. Se producen cuando hay al menos una incisión, realizada en un centro quirúrgico, a través de la piel, la membrana mucosa o una incisión que se dejó abierta durante un procedimiento quirúrgico anterior^(1)^.

Presentan una incidencia variable y representan un gran desafío en la prevención de infecciones relacionadas con la atención a la salud (IRAS), ocupando el tercer lugar entre todas las infecciones en los servicios de salud y comprendiendo, en Brasil, del 14% al 16% de las IRAS encontradas en pacientes hospitalizados^(2)^.

En países de altos ingresos, como los Estados Unidos de América, esta cuestión también sigue siendo relevante. Datos recientes indican que las ISQ son responsables de más de dos millones de infecciones nosocomiales en pacientes hospitalizados, lo que corresponde al 20% de las principales infecciones, lo que pone de manifiesto la gravedad del problema incluso en sistemas de salud avanzados^(3-4)^. En Europa, la situación no es diferente, ya que se considera la segunda infección más recurrente, responsable de unos 500 000 casos al año^(5)^.

El aumento del tiempo de hospitalización, hasta tres veces^(5-6)^, el aumento de los costos hospitalarios y el riesgo de muerte de 2 a 11 veces mayor en comparación con los pacientes que no desarrollan ISQ^(3,7-8)^ son impactos significativos que no solo sobrecargan los sistemas hospitalarios, sino que también aumentan los costos operativos, limitan la disponibilidad de camas para otros pacientes y exigen atención prioritaria en el contexto de la asistencia sanitaria^(9-10)^.

Estos factores no solo afectan a los hospitales, sino también a los propios pacientes, que se enfrentan a un mayor sufrimiento, complicaciones postoperatorias e impactos socioeconómicos, como el alejamiento prolongado del trabajo^(7)^. A la luz de estos hechos, la actuación de un equipo multidisciplinario es crucial para la implementación de estrategias rigurosas de vigilancia, control de infecciones y mejora de las prácticas quirúrgicas, siendo, por lo tanto, esenciales para mitigar estos riesgos y garantizar mejores resultados clínicos^(11)^.

Ante los impactos presentados y considerando su evitabilidad, es imprescindible implementar medidas de prevención para mitigar los riesgos de que se produzca este evento en las instituciones de salud, de acuerdo con las directrices internacionales de la Organización Mundial de la Salud (OMS) y las nacionales de la Agencia de Vigilancia Sanitaria (ANVISA).

La implementación de estas estrategias refleja un compromiso global con la seguridad del paciente y la mejora continua de la atención quirúrgica, ya que estas medidas no solo tienen como objetivo reducir las tasas de ISQ, sino también minimizar los riesgos de complicaciones graves, garantizando la calidad de la atención y promoviendo procedimientos quirúrgicos más seguros.

En este contexto, el enfermero desempeña un papel destacado, ya que este profesional es responsable de la planificación y la implementación de los cuidados de enfermería necesarios y de la educación en salud. Además, debe tener conocimientos sobre las complicaciones, orientados a su prevención y detección precoz, contribuyendo de manera eficaz al bienestar del paciente y a su nueva condición de vida^(12)^.

Por lo tanto, es imperativo que tengamos un conocimiento sólido sobre la relación de los factores de riesgo con el resultado de la ISQ, ya que estos factores se consideran sustitutos de la causa subyacente y se utilizan para predecir resultados. Cuando un factor se asocia repetidamente con la ISQ, se convierte en un indicador más confiable para el desarrollo de estrategias de prevención y vigilancia. Además, los factores de riesgo consistentes pueden utilizarse para estandarizar las tasas de ISQ entre los pacientes, lo que permite comparaciones más precisas y contribuye a las iniciativas de mejora de la calidad^(7)^.

Ante lo expuesto, los estudios con un análisis exhaustivo de una gran cohorte de pacientes, junto con la identificación de factores de riesgo y protección, aumentan nuestra comprensión de esta compleja complicación quirúrgica. Comprender y estratificar este riesgo es esencial para mejorar los resultados, orientar las decisiones de vigilancia y asignar los recursos de manera eficiente, ya que estas tasas en sí mismas son difíciles de interpretar sin una estratificación del riesgo^(7,13)^. Por lo tanto, una herramienta de estratificación del riesgo es un elemento clave en el proceso diagnóstico y permite identificar de forma anticipada a las personas que presentan factores de riesgo elevados, lo que permite implementar estrategias de monitoreo y prevención más eficaces, con el objetivo de mejorar continuamente la calidad y la seguridad del paciente.

En este contexto, el objetivo de este estudio es identificar los factores de riesgo de infección del sitio quirúrgico en cirugías realizadas en un hospital universitario y establecer un modelo de predicción.

## MÉTODO

### Tipo de Estudio

Se trata de una investigación de enfoque cuantitativo, observacional, de tipo cohorte retrospectiva.

### Lugar de Recolección de Datos

El lugar elegido para desarrollar el estudio fue el Hospital Universitario Federal, referencia en el tratamiento de diversas patologías de alta complejidad e importante centro de asistencia, enseñanza, investigación y extensión, ubicado en Río de Janeiro.

### Muestra del Estudio

La muestra utilizada en la investigación se extrajo de la base de datos de vigilancia quirúrgica, perteneciente a la Coordinación de Control de Infecciones Hospitalarias (CCIH), interconectada con el sistema de historias clínicas electrónicas del hospital, que abarca el periodo de 2009 a 2019. El periodo temporal adoptado en esta investigación se definió con el fin de garantizar la coherencia y la comparabilidad de los datos. Se optó por excluir los años 2020 y 2021 debido al impacto directo de la pandemia de COVID-19 sobre los flujos quirúrgicos y la dinámica hospitalaria. Durante este periodo, se produjo una reducción significativa en la realización de procedimientos electivos, se dio prioridad a las cirugías urgentes y de emergencia, y se llevaron a cabo reestructuraciones en los servicios de salud para hacer frente a la crisis sanitaria.

Estos cambios comprometieron la regularidad de las prácticas quirúrgicas y los procesos de notificación y vigilancia de las infecciones relacionadas con la asistencia sanitaria, especialmente en lo que se refiere a la ISQ. Como consecuencia, los datos de esos años presentan características atípicas, que podrían introducir sesgos en el análisis estadístico y perjudicar la identificación de patrones epidemiológicos y factores de riesgo en contextos asistenciales estables. La investigación incluyó 20 778 cirugías.

### Criterios de Selección

Se incluyeron en el estudio los datos de las cirugías que cumplían los criterios establecidos por la Agencia Nacional de Vigilancia Sanitaria (ANVISA): aquellas cirugías en las que la fecha de alta hospitalaria del paciente difería de la fecha de la intervención y aquellas en las que los pacientes tenían 18 años o más.

Se excluyeron las reoperaciones realizadas en un intervalo inferior a 30 días después de la primera intervención, las cirugías realizadas en hemodinámica y los procedimientos quirúrgicos indicados para el tratamiento de traumatismos agudos.

### Recolección de Datos

La recolección de datos se llevó a cabo entre octubre de 2023 y abril de 2024.

### Análisis de Datos

Se analizaron las infecciones del sitio quirúrgico en los procedimientos quirúrgicos y sus posibles factores de riesgo. La categorización de la ISQ se basó en la ausencia y la presencia de infección, que podía ser incisional, superficial o profunda, de órganos y espacios.

Como variables independientes se consideraron los factores de riesgo de ISQ, aquellos cuya presencia se asocia con una mayor probabilidad de que se desarrolle una infección: sexo; potencial de contaminación de la herida quirúrgica (limpia, potencialmente contaminada, contaminada e infectada); condiciones clínicas del paciente en el preoperatorio, analizadas por ASA I, II, III, IV y V, criterio propuesto por la *American Society of Anesthesiologists*; urgencia (no y si); implante (no y si); tipo de procedimiento quirúrgico; y duración de la cirugía.

Los análisis se realizaron mediante estadística descriptiva, con cálculo de la media, la mediana y la desviación estándar (DE) para las variables cuantitativas. Las variables cualitativas se describieron en función de la frecuencia absoluta (n) y relativa (%). Los análisis bivariados que incluyeron variables cualitativas o categóricas comprendieron la prueba de chi cuadrado y la razón de posibilidades.

Para los análisis bivariados, la normalidad de las variables cuantitativas o continuas se evaluó primero mediante la prueba de Shapiro-Wilk. Dado que los datos no presentaban una distribución normal, se utilizó la prueba no paramétrica U de Mann-Whitney para verificar la asociación entre las variables demográficas y clínicas y el resultado.

En el análisis de regresión logística multivariante se utilizó el método backward, es decir, en cada paso se retiraron del modelo las variables que no se asociaron significativamente con el desenlace. Así, al final del modelo multivariante, permanecieron aquellas variables significativamente asociadas con la presencia de infección posquirúrgica.

De manera similar, se verificó, mediante análisis bivariados, la asociación entre las variables demográficas y clínicas y la infección hospitalaria. Para los análisis se asumió un nivel de significación del 5% (p ≤ 0,05) y un intervalo de confianza del 95%. Todos los análisis se realizaron con el programa IBM SPSS (v.16.0) y se utilizó el programa Microsoft Excel para elaborar las figuras.

### Aspectos Éticos

El estudio obtuvo la aprobación del Comité de Ética en Investigación (CEP), con el dictamen n.º 6.231.690, de conformidad con la Resolución n.º 466/2012 del Consejo Nacional de Salud (CNS). Al tratarse de un estudio retrospectivo realizado a partir de fuentes secundarias inherentes al proceso de vigilancia y control de la LRA ya realizado por el CCIH del lugar de estudio, se concedió la exención de la firma del Término de Consentimiento Libre y Esclarecido para realizar el estudio.

## RESULTADOS

El estudio incluyó el análisis de 20 778 cirugías electivas y de urgencia, distribuidas a lo largo de 10 años de contabilización. Se observó que los años 2013 (n = 2119; 10,2%) y 2016 (n = 2106; 10,1%) fueron los que concentraron la mayor prevalencia de cirugías realizadas.

La mayoría de los procedimientos se realizaron en pacientes de sexo femenino (58,8%). La edad de los pacientes varió de 18 a 97 años, con una media de 52,0 años (desviación estándar = 16,3 años) y una mayor prevalencia de eventos quirúrgicos (23,0%) en el grupo de edad de 51 a 60 años (Tabla 1).

**Tabla 1:** Análisis descriptivos de las variables demográficas y clínicas de los pacientes sometidos a procedimientos quirúrgicos entre los años 2009 y 2019 (N = 20778) – Río de Janeiro, RJ, Brasil, 2023–2024.

Variables	N	%	Min-Max	Media (±DE)
Sexo				
Masculino	8622	41,5		
Femenino	12156	58,5		
Edad			18–97	52,0 (±16,3)
Grupos de edad				
De 18 a 30 años	2551	12,3		
31 a 40 años	2746	13,2		
41 a 50 años	3819	18,4		
51 a 60 años	4875	23,5		
61 a 70 años	4017	19,3		
71 o más	2770	13,3		
Duración en horas			0,083–23,3	2,5 (±1,6)
ASA				
I	6273	30,2		
II	10375	49,9		
III	3818	18,4		
IV	305	1,5		
V	6	0,0		
Tipo de cirugía				
Electiva	19390	93,3		
Urgencia	1388	6,7		
Resultado				
Óbito	634	3,1		
Alta	20144	96,9		
Uso de video				
No	17607	84,7		
Si	3171	15,3		
Utilizó implantes				
No	15714	75,6		
Si	5064	24,4		

Notas: DE = desviación estándar; ASA = *American Society of Anesthesiologists*.

En los hombres, la edad media fue de 53,03 (desviación estándar = 16,90) y en las mujeres fue de 51,29 (desviación estándar = 15,78). El análisis de varianza para el sexo en relación con las edades, mediante la técnica de Mann-Whitney, presentó una violación de la hipótesis nula (U = 5,62 × 10^7^; p < 0,001; VS-MPR = 8,51 × 10^15^; rbs = 0,07), lo que indica patrones distintos de variación de la edad entre los sexos.

El cálculo de la duración de las cirugías realizadas mostró que, en promedio, se dedicaron 2,46 horas (±1,60). La mayoría de los procedimientos fueron de carácter electivo (93,3%) y no requirieron reintervención (97,6%), mientras que el 6,7% fueron cirugías de urgencia. Otra característica observada fue la ASA, cuya clasificación más frecuente fue la II (49,9%). El análisis de los resultados clínicos reveló que el 96,9% (n = 20 144) de los pacientes fueron dados de alta del hospital, mientras que el 3,1% (n = 634) fallecieron. (Tabla 1)

El uso de implantes se registró en el 24,4% (n = 5064) de los procedimientos, lo que refuerza la importancia de estos dispositivos en el manejo quirúrgico, mientras que el 15,3% (n = 3171) de las cirugías utilizaron videocirugía.

En la muestra del estudio, se registraron 1491 casos de infección del sitio quirúrgico, lo que supone una tasa de incidencia de ISQ de aproximadamente el 7,2%. De estas, la mayoría (54,59%) fueron superficiales y el 31,52% intracavitarias. El lugar de identificación de la infección fue, en gran parte, en los ambulatorios de egresados (54,19%), según se muestra en la Tabla 2.

**Tabla 2:** Caracterización de la incidencia de infección del sitio quirúrgico en los participantes a lo largo de 10 años de observación (N = 20 778) – Río de Janeiro, RJ, Brasil, 2023–2024.

Variables	N	%
Ocurrencia de infección		
Si	1,491	7,18
No	19.287	92,82
Tipo de infección		
Superficial	814	54,59
Intracavitaria	470	31,52
Profunda	207	13,88
Locales de diagnóstico de la infección		
Ambulatorio	808	54,19
Sala de internación	683	45,81

En cuanto al sexo, no hubo diferencias estadísticamente significativas en la incidencia de infección entre hombres (7,0%) y mujeres (7,4%) (OR = 1,05; IC del 95%: 0,94–1,17; p = 0,374). La evaluación del grupo de edad, por su parte, reveló una asociación significativa con la presencia de infección (p < 0,0001). Se observó que, con el avance de la edad, la probabilidad del desenlace es mayor en los grupos de mayor edad (a partir de 41-50 años) en comparación con los más jóvenes (18–30 años), como se muestra en la Tabla 3.

**Tabla 3:** Tipos de servicios quirúrgicos y factores asociados a la infección del sitio quirúrgico a lo largo de 10 años de observación (N = 20778) – Río de Janeiro, RJ, Brasil, 2023–2024.

Características estudiadas	Presencia de infección				OR (IC95%)	p
	No		Si			
	n	%	n	%		
Sexo						
Masculino	11300	93,0	856	7,0	1,05 (0,94–1,17)	0,374
Femenino	7987	92,6	635	7,4		
Grupos de edad						
De 18 a 30 años	2442	95,7	109	4,3	1,0	
31 a 40 años	2616	95,3	130	4,7	1,11 (0,86–1,45)	0,419
41 a 50 años	3576	93,6	243	6,4	1,52 (1,21–1,92)	< 0,0001
51 a 60 años	4518	92,7	357	7,3	1,77 (1,42–2,21)	< 0,0001
61 a 70 años	3655	91,0	362	9,0	2,22 (1,78–2,77)	< 0,0001
71 o más	2480	89,5	290	10,5	2,62 (2,09–3,29)	< 0,0001
Tipo de cirugía						
Urgencia	1209	87,1	179	12,9	2,04 (1,73–2,41)	< 0,0001
Electiva	18078	93,2	1312	6,8	1,0	
ASA						
Clasificación I	6045	96,4	2299	3,6	1,0	
Clasificación II	9629	92,8	746	7,2	2,04 (1,76–2,38)	< 0,0001
Clasificación III	3340	87,5	478	12,5	3,77 (3,20–4,44)	< 0,0001
Potencial de contaminación						
Limpia	11130	94,7	626	5,3	1,0	
Potencialmente contaminada	5955	92,9	457	7,1	1,37 (1,21–1,55)	< 0,0001
Contaminada	1726	84,2	323	15,8	3,33 (2,88–3,84)	< 0,0001
Infectada	476	84,8	85	15,2	3,18 (2,49–4,01)	< 0,0001
Uso de video						
No	16245	92,3	1362	7,7	1,98 (1,64–2,38)	< 0,0001
Si	3042	95,9	129	4,1	1,0	
Uso de implantes						
No	13379	92,1	1146	7,9	1,47 (1,30–1,66)	< 0,0001
Si	5908	94,5	345	5,5	1,0	
Otorrinolaringología						
Si	1017	95,9	43	4,1	0,53 (0,38–0,72)	< 0,0001
No	18270	92,7	1448	7,3	1,0	
Neurocirugía						
Si	1215	92,5	99	7,5	1,06 (0,86–1,31)	0,603
No	18072	92,8	1392	7,2	1,0	
Urología						
Si	1255	93,7	85	6,3	0,87 (0,69–1,09)	0,122
No	18032	92,8	1406	7,2	1,0	
Ginecología						
Si	1507	92,1	129	7,9	1,12 (0,93–1,35)	0,247
No	17780	92,9	1362	7,1	1,0	
Cirugía Plástica						
Si	1684	96,6	60	3,4	0,44 (0,34–0,57)	< 0,0001
No	17603	92,5	1431	7,5	1,0	
Ortopedia y trauma						
Si	3329	95,8	147	4,2	0,52 (0,44–0,62)	< 0,0001
No	15958	92,2	1344	7,8	1,0	
Cirugía General						
Si	7246	91,9	638	8,1	1,25 (1,12–1,38)	< 0,0001
No	12041	93,4	853	6,6	1,0	

Notas: OR = odds ratio; IC = intervalo de confianza; ASA = *American Society of Anesthesiologists*.

La comparación de los pacientes de entre 31 y 40 años con el grupo más joven no reveló una asociación significativa con el desenlace (OR = 1,11; IC del 95%: 0,86-1,45). Sin embargo, para el siguiente grupo de edad, el riesgo de ISQ fue 1,52 veces mayor en relación con el mismo grupo de referencia (IC95%:1,21–1,92). En resumen, se observó un aumento en la probabilidad de infección con el aumento de la edad.

El potencial de contaminación de la cirugía se evaluó en cuatro categorías, utilizando la clasificación de cirugía limpia como referencia. En comparación con esta, se observó que los pacientes sometidos a procedimientos potencialmente contaminados tenían un 37% más de probabilidades de desarrollar una infección. Para los procedimientos contaminados, el riesgo fue 3,33 veces mayor (IC95%: 2,88–3,84). En los procedimientos infectados, la probabilidad de desarrollar la complicación fue 3,18 veces mayor (IC95%: 2,49–4.01).

Las personas expuestas a cirugías de urgencia tuvieron 2,04 veces más probabilidades (IC 95%: 1,73–2,41) de presentar infección en comparación con las sometidas a cirugías electivas. El análisis de la puntuación ASA también reveló un fuerte gradiente de riesgo, utilizando ASA I como categoría de referencia. Los pacientes clasificados como ASA II tuvieron el doble de riesgo de infección (OR = 2,04; IC del 95%: 1,76–2,38), mientras que para ASA III, el riesgo fue casi cuatro veces mayor (OR = 3,77; IC del 95%: 3,20–4,44).

El uso de la videolaparoscopia se asoció con un menor riesgo de infección, con una tasa del 4,1% en los procedimientos por vídeo frente al 7,7% en las cirugías convencionales (OR = 1,98; IC del 95%: 1,64–2,38; p < 0,0001). El uso de implantes también se mostró como un factor protector, ya que los pacientes sometidos a procedimientos sin implantes presentaron una tasa de infección del 7,9%, mientras que los que utilizaron implantes tuvieron una incidencia del 5,5% (OR = 1,47; IC del 95%: 1,30–1.66; p < 0,0001).

El análisis de la asociación por especialidad quirúrgica (Tabla 3) reveló que los servicios de cirugía plástica (OR = 0,44; IC del 95%: 0,34-0,57), ortopedia (OR = 0,52; IC 95%: 0,44–0,62) y otorrinolaringología (OR = 0,53; IC 95%: 0,38–0,72) presentaban una probabilidad significativamente menor de infección del sitio quirúrgico en comparación con los pacientes que se sometieron a procedimientos en otros servicios. En el caso del servicio de urología, aunque se observó una tendencia a un menor riesgo relativo, la asociación no alcanzó significación estadística (RR = 0,87; IC del 95%: 0,69–1,09; p = 0,122).

En contraste con las especialidades que actuaron como factor de protección, la cirugía general se presentó como un factor de riesgo para el desenlace. Los pacientes de este servicio tuvieron un riesgo 25% mayor de desarrollar infección (OR = 1,25; IC del 95%: 1,12–1,38) en comparación con los demás.

Los pacientes que desarrollaron ISQ tuvieron un tiempo medio de cirugía mayor (3,43 h ± 1,96 h) en comparación con los que no desarrollaron infección (2,38 h ± 1,54 h). En promedio, la duración del procedimiento de los que presentaron infección fue, en promedio, 1,05 h más larga en comparación con los que no tuvieron infección. Las medianas difirieron en 1 h más para las cirugías cuyos pacientes desarrollaron infección (p < 0,0001), lo que sugiere una fuerte asociación entre la mayor duración del procedimiento y la presencia de infección posoperatoria (Tabla 4).

**Tabla 4:** Asociación entre el tiempo medio (en horas) de cirugía y la infección del sitio quirúrgico a lo largo de 10 años de observación (N = 20778) – Río de Janeiro, RJ, Brasil, 2023–2024.

	N	Media en horas (±DE)	Min-Max	Mediana	IIC 25%–75%	p^*^
Infección						
Si	1491	3,43 (1,96)	0,33–13,98	3,00	2,00–4,50	< 0,0001
No	19287	2,38 (1,54)	0,08–23,25	2,00	1,25–3,00	

Notas: IIC = Intervalo Intercuartil; *test U de Mann-Whitney.

La Tabla 5 presenta los resultados del análisis de regresión logística multivariante, en el que el tipo de cirugía y el uso de implantes no se asociaron significativamente con el resultado en el modelo multivariante. Se mantuvieron como factores potenciales de riesgo de infección en esta población la edad, la duración de la intervención quirúrgica, las intervenciones en las que no se utilizó vídeo, las intervenciones potencialmente contaminadas, contaminadas e infectadas, la exposición al servicio de cirugía general y las clasificaciones ASA II y III.

**Tabla 5:** Análisis de regresión logística multivariante entre las características relacionadas con la presencia de infección del sitio quirúrgico a lo largo de 10 años de observación – Río de Janeiro, RJ, Brasil, 2023–2024.

	OR	IC95%	*p*
Modelo 1			
Edad (años)	1,01	1,01–1,02	< 0,0001
Duración del procedimiento (horas)	1,34	1,30–1,38	< 0,0001
Cirugía de urgencia (*vs.* electiva)	1,02	0,83–1,26	0,835
Sin uso de video (*vs.* con uso de video)	2,73	2,23–3,36	< 0,0001
Uso de implantes	1,05	0,91–1,21	0,537
Potencialmente contaminada (*vs* limpia)	1,50	1,23–1,72	< 0,0001
Contaminada (*vs* limpia)	2,94	2,49–3,47	< 0,0001
Infectada (*vs* limpia)	2,68	2,00–3,61	< 0,0001
Cirugía general (*vs.* los otros servicios médicos)	1,67	1,48–1,89	< 0,0001
ASA II (*vs.* ASA I)	1,45	1,23–1,72	< 0,0001
ASA III (*vs.* ASA I)	1,89	1,56–2,29	< 0,0001
Modelo 2			
Edad (años)	1,01	1,00–1,01	< 0,0001
Duración del procedimiento (horas)	1,34	1,30–1,38	< 0,0001
Sin uso de video (*vs.* con uso de video)	2,74	2,23–3,36	< 0,0001
Uso de implantes	1,05	0,91–1,21	0,531
Potencialmente contaminada (*vs* limpia)	1,50	1,30–1,72	< 0,0001
Contaminada (*vs* limpia)	2,95	2,50–3,58	< 0,0001
Infectada (*vs* limpia)	1,68	1,49–1,89	< 0,0001
Cirugía general (*vs.* los otros servicios médicos)	1,68	1,49–1,89	< 0,0001
ASA II (*vs.* ASA I)	1,45	1,23–1,72	< 0,0001
ASA III (*vs.* ASA I)	1,90	1,57–2,29	< 0,0001
Modelo 3			
Edad (años)	1,01	1,01–1,02	< 0,0001
Duración del procedimiento (horas)	1,34	1,30–1,38	< 0,0001
Sin uso de video (*vs.* con uso de video)	2,72	2,22–3,34	< 0,0001
Potencialmente contaminada (*vs* limpia)	1,52	1,33–1,73	< 0,0001
Contaminada (*vs* limpia)	3,00	2,57–3,50	< 0,0001
Infectada (*vs* limpia)	2,76	2,10–3,63	< 0,0001
Cirugía general (*vs.* los otros servicios médicos)	1,68	1,48–1,89	< 0,0001
ASA II (*vs.* ASA I)	1,46	1,24–1,72	< 0,0001
ASA III (*vs.* ASA I)	1,91	1,58–2,30	< 0,0001

Notas: OR = Odds Ratio; IC = Intervalo de Confianza; ASA = *American Society of Anesthesiologists*; *vs*. = *versus*.

En el modelo final (modelo 3) de la regresión logística, la edad se asoció con un aumento del 1% en la razón de probabilidad del evento por cada año adicional (OR = 1,01; IC del 95%: 1,01–1,02). Por otro lado, la razón de probabilidad observada para la duración del procedimiento evidenció que, por cada hora adicional de ejecución, aumentaba en un 34% la probabilidad de infección (OR = 1,34; IC del 95%: 1,30–1,38). Los procedimientos que no utilizaron video también se asociaron con una mayor probabilidad de infección, con un incremento del 172% en la razón de probabilidad del evento (OR = 2,72; IC del 95%: 2,22–3,34), lo que sugiere que el uso de video en procedimientos quirúrgicos puede ser un factor de protección potencial para la ISQ.

También se investigó el potencial de contaminación y se observó que las cirugías clasificadas como potencialmente contaminadas (OR = 1,52; IC del 95%: 1,33–1,73), contaminadas (OR = 3,00; IC95%: 2,57–3,50) e infectadas (OR = 2,76; IC95%: 2,10–3,63) se asociaron significativamente con la presencia de infección. La cirugía general, en comparación con los demás servicios médicos, mostró un aumento del 68% (OR = 1,68; IC del 95%: 1,48–1,89) en la probabilidad de infección. Por último, los individuos clasificados tanto en la categoría ASA II (OR = 1,46; IC 95%: 1,24–1,72) como en la categoría ASA IV (OR = 1,91; IC 95%: 1,58–2,30) presentaron una mayor probabilidad de infección en comparación con los clasificados como ASA I.

## DISCUSIÓN

En las últimas décadas, se ha observado un aumento progresivo en el número de intervenciones quirúrgicas, lo que intensifica la preocupación por las ISQ, cuyas complicaciones generan impactos tanto financieros como sociales^9^. Este fenómeno es el resultado de una interacción compleja y multifactorial entre factores extrínsecos e intrínsecos, que confiere a cada individuo un riesgo específico de desarrollar infecciones^(12)^.

La identificación de los factores de riesgo y la construcción de un modelo predictivo de riesgo para la aparición de ISQ contribuyen a la planificación y adopción de estrategias de prevención y vigilancia, lo que implica un enfoque amplio, siendo el enfermero uno de los principales profesionales de la salud que debe participar con rigor en todas las fases de la atención quirúrgica.

El análisis sociodemográfico de este estudio reveló que la mayoría de los procedimientos se realizaron en pacientes de sexo femenino. Este resultado está en consonancia con investigaciones anteriores y puede estar asociado al hecho de que las mujeres tienden a acudir más a los servicios de salud, especialmente para medidas preventivas y seguimiento médico^(14)^.

En relación con la variable sexo, esta investigación no encontró diferencias estadísticamente significativas en la incidencia de infección del sitio quirúrgico entre hombres y mujeres. Este resultado concuerda con otros hallazgos, como los de un sólido estudio de cohorte multicéntrico con 17 353 pacientes sometidos a cirugía gastrointestinal^(15)^, en el que, aunque la incidencia de esta complicación fue ligeramente mayor en los hombres, el análisis estadístico no demostró ninguna asociación (p = 0,088).

Por lo tanto, la variable sexo no se consideró para integrar el modelo aquí propuesto. La edad es un factor de riesgo ampliamente reconocido tanto para el compromiso de la cicatrización de heridas como para el desarrollo de infecciones del sitio quirúrgico (ISQ).

Los pacientes de más edad tienden a presentar un mayor número de comorbilidades y alteraciones fisiológicas^(16)^, lo que puede inclinar a una mayor vulnerabilidad a la infección. Este escenario fue corroborado por un estudio retrospectivo con pacientes ortopédicos, en el que el aumento de la edad se destacó como un predictor significativo para la aparición de ISQ^(16–19)^.

De manera similar, un metaanálisis que investigó los factores de riesgo de infección posoperatoria en pacientes oncológicos evidenció que la franja de edad superior a 60 años está significativamente asociada a un mayor riesgo de ISQ^(20)^. Corroborando estos hallazgos, el presente estudio también demostró una asociación significativa entre la edad avanzada y la incidencia de ISQ, lo que indica un aumento progresivo del riesgo en los grupos de edad más elevados. Este resultado destaca la necesidad de estrategias de monitoreo riguroso en pacientes de edad avanzada sometidos a procedimientos quirúrgicos, con el fin de minimizar las complicaciones infecciosas y optimizar los resultados clínicos.

Los resultados de la investigación resaltan que la cirugía de urgencia se asoció significativamente con una mayor probabilidad de infección del sitio quirúrgico (ISQ), demostrando que la cirugía de urgencia duplicó la probabilidad de ISQ en comparación con los procedimientos electivos, lo que corrobora hallazgos previos en la literatura^(19)^. Estos hallazgos advierten de la importancia de las estrategias preventivas y de la vigilancia intensificada en escenarios en los que la intervención quirúrgica se ha realizado con carácter de urgencia. Sin embargo, tras realizar la regresión logística, este no fue un factor de riesgo a tener en cuenta en el modelo propuesto en esta investigación.

En un estudio prospectivo que involucró a 100 pacientes de subespecialidades quirúrgicas, realizado durante un período de 6 meses, se observó que las tasas de ISQ aumentaban progresivamente a medida que la clasificación ASA se elevaba, siendo especialmente más altas en pacientes clasificados como ASA III y IV^(19)^, hallazgo similar al identificado en esta investigación. Este resultado está en consonancia con las evidencias descritas previamente en la literatura y con el índice de riesgo de ISQ del CDC^(21–23)^, que ya había señalado a la ASA como uno de los tres principales factores de riesgo para el ISQ, asociación que fue posteriormente corroborada por el modelo predictivo propuesto en este estudio.

Los avances tecnológicos en las técnicas quirúrgicas, como el uso de equipos de video, han revolucionado la práctica quirúrgica en las últimas décadas y, en este estudio, al igual que en la literatura, han demostrado un efecto protector contra la ISQ^(24–25)^. Las cirugías clasificadas como contaminadas e infectadas tienen más probabilidades de evolucionar hacia una ISQ en comparación con las cirugías clasificadas como limpias por su potencial de contaminación, según los resultados de esta investigación y corroborado por estudios anteriores^(15,20)^.

La duración prolongada de la intervención quirúrgica constituye un factor de riesgo significativo para la infección del sitio quirúrgico^(26)^. La duración prolongada de la cirugía suele reflejar una mayor complejidad del procedimiento, retos técnicos adicionales y una exposición prolongada de los tejidos, elementos que, en conjunto, crean condiciones favorables para el desarrollo de complicaciones infecciosas^(16,27–28)^, lo que sugiere que una planificación quirúrgica adecuada para reducir la duración de la cirugía puede ayudar a reducir el riesgo de ISQ.

El uso de implantes, en esta investigación, se comportó como un factor protector para la ISQ en los análisis estadísticos, un hallazgo que concuerda con la literatura disponible ^(29)^. Sin embargo, es importante destacar que esta variable no se mantuvo como predictor independiente en el modelo final de regresión logística, lo que podría sugerir que su aparente efecto protector podría estar asociado a otros factores de confusión no controlados en el análisis.

En cuanto a los resultados aquí presentados, cabe destacar que los procedimientos realizados por la cirugía general están significativamente asociados a un mayor riesgo relativo de ISQ en comparación con otras especialidades, lo que constituye un factor predictivo importante en la composición del modelo predictivo. Sin embargo, a pesar de la consistencia interna de los datos, la comparación con otros estudios es limitada porque gran parte de la evidencia disponible tiene como objetivo la construcción de modelos predictivos específicos para poblaciones sometidas al mismo tipo de procedimiento. Por ejemplo, los estudios sobre cirugía colorrectal suelen evaluar riesgos como la preparación intestinal y la profilaxis antibiótica, mientras que las investigaciones que abordan otros procedimientos gastrointestinales^(15,30)^ se centran en factores de riesgo relacionados con la técnica quirúrgica y el paciente.

Este enfoque fragmentado por procedimientos genera modelos de riesgo no generalizables, lo que dificulta su aplicación en hospitales de atención generalista, que abarcan una diversidad de técnicas y especialidades quirúrgicas.

Este estudio presenta limitaciones inherentes al diseño retrospectivo y al uso de datos secundarios, que pueden estar sujetos a subnotificación y variabilidad en la calidad de los registros. Además, se llevó a cabo con datos de un solo hospital, lo que puede restringir la generalización de los hallazgos a otras realidades institucionales.

A pesar de estas limitaciones, los resultados contribuyen significativamente al área de la enfermería y la salud, al ofrecer evidencia epidemiológica sólida sobre los factores asociados a la ISQ. La identificación de variables independientes asociadas al desenlace infeccioso sustenta el desarrollo de modelos predictivos de riesgo, con potencial aplicación en la estratificación y priorización de pacientes en el ámbito de la vigilancia activa.

## CONCLUSIÓN

Este estudio establece un modelo predictivo para la aparición de infección del sitio quirúrgico basado en seis variables asociadas al resultado estudiado y de fácil obtención clínica, tales como: edad, duración del procedimiento, uso de video, potencial de contaminación, puntuación ASA y servicio de cirugía general.

La principal contribución de este modelo reside en su amplia aplicabilidad, ya que utiliza parámetros universalmente medibles. Esto supera una barrera común de los modelos más complejos, que a menudo dependen de marcadores de laboratorio específicos y dificultan la comparación entre diferentes contextos quirúrgicos.

En la práctica, el modelo se traduce en una herramienta eficaz para una posible estratificación del riesgo, lo que permite a los equipos de salud dirigir las estrategias preventivas de forma más asertiva, optimizar el uso de los recursos y, fundamentalmente, fortalecer la seguridad del paciente. Cabe destacar también la importancia de elaborar nuevos estudios con enfoques metodológicos estandarizados, con el fin de permitir análisis transversales más sólidos y mejorar la identificación del riesgo en escenarios quirúrgicos heterogéneos.

Así, la capacidad de identificar perfiles de pacientes con mayor riesgo de ISQ representa un avance significativo en la planificación de una gestión predictiva del riesgo y en una vigilancia más dirigida al riesgo identificado. Este aspecto es particularmente relevante en hospitales de alta complejidad, donde puede generar impactos sustanciales en la calidad de la atención y en los resultados clínicos.

Cabe destacar como limitación importante del estudio el hecho de que el modelo se haya desarrollado con datos de un solo centro hospitalario, lo que enfatiza la necesidad de validarlo en múltiples escenarios institucionales, preferiblemente en hospitales con diferentes perfiles de complejidad y características epidemiológicas distintas. Sin embargo, el modelo predictivo propuesto en el estudio aporta contribuciones valiosas para la mejora de las prácticas de prevención de ISQ y para orientar futuras investigaciones sobre el tema de la seguridad del paciente.

## Data Availability

Todo el conjunto de datos que apoya los resultados de este estudio fue publicado en el propio artículo.
